# Investigating Australian Consumers’ Perceptions of and Preferences for Different Styles of Sparkling Wine Using the Fine Wine Instrument

**DOI:** 10.3390/foods10030488

**Published:** 2021-02-24

**Authors:** Naomi Verdonk, Renata Ristic, Julie A. Culbert, Karma Pearce, Kerry L. Wilkinson

**Affiliations:** 1 School of Agriculture, Food and Wine, Waite Research Institute, The University of Adelaide, Waite Campus, PMB 1, Glen Osmond, SA 5064, Australia; naomi.verdonk@adelaide.edu.au (N.V.); renata.ristic@adelaide.edu.au (R.R.); julie.culbert@adelaide.edu.au (J.A.C.); 2 School of Pharmacy and Medical Sciences, University of South Australia, GPO Box 2471, Adelaide, SA 5001, Australia; karma.pearce@unisa.edu.au

**Keywords:** Champagne, descriptive analysis, hedonic liking, Moscato, Prosecco, segmentation

## Abstract

This study investigated consumer preferences for different styles of sparkling wine and the influence of wine style and occasion on sparkling wine purchasing and consumption behavior. Australian consumers (*n* = 203) completed an online survey and blind tasting of representative styles of commercial sparkling wines, including Champagne. Wine sensory profiles were determined by descriptive analysis using a trained panel (*n* = 12) and consumers were segmented into ‘No Frills’, ‘Aspirant’ and ‘Enthusiast’ clusters using the Fine Wine Instrument. Consumer perceptions, preferences and liking were measured using 9-point hedonic scales and compared via statistical analysis. Consumers anticipated liking Champagne and sparkling white wine the most, and Moscato and Prosecco the least, but on tasting, could only readily identify the Moscato and sparkling red wines, as the most contrasting wine styles. As such, liking scores for the Champagne and sparkling white wine were significantly lower based on tasting (median scores were 6.0, compared with 9.0 and 8.0 for survey responses, respectively). Consumers’ preconceived expectations of different sparkling wine styles clearly influenced purchasing and consumption behavior. Aspirants and Enthusiasts were more likely to spend more per bottle for Champagne and sparkling white wine, and consumption of these sparkling wines was most frequently associated with celebratory occasions, such as anniversaries, birthdays, Christmas, New Year and weddings.

## 1. Introduction

Australia is among the top ten producers of sparkling wine (by volume) in the world, producing ~7 million cases/annum [1], and almost half of Australia’s adult population (i.e., ~9 million consumers) regularly enjoy this fine wine style [1]. Sparkling white wine accounts for the ‘lion’s share’ of Australian sparkling wine production, but sparkling rosé, sparkling red, and increasingly, Prosecco and Moscato, are also produced in Australia [2]. Domestic sparkling wine sales have remained relatively constant in Australia, whereas the volume and value of sparkling wine being imported (predominantly Champagne) is growing, while exports are declining [1]. 

Previous studies have demonstrated significant diversity in the sensory profiles of Australian sparkling white and Moscato wines [3,4]. For sparkling white wines, variation in sensory qualities can be attributed to the method of production; carbonated and Charmat wines are typically fruit-driven styles of sparkling wine, whereas transfer and Méthode Traditionelle wines exhibit complexity (e.g., yeasty, toasty, bready characters) due to a combination of bottle fermentation, aging with lees contact and/or yeast autolysis [5,6]. Within the domestic (Australian) sparkling wine market, there are consumer segments with different preferences for these different styles of sparkling wine [3,4,7]. Wine sensory properties are amongst the most important factors influencing consumer preference [8,9,10]. However, consumers tend to find sparkling wine more difficult to evaluate than table wine, especially less involved (‘novice’) consumers [11,12]. Younger and/or less involved consumers tend to prefer sweeter, fruitier styles of wine [13], then as consumer involvement increases, preferences transition from sweet to dry, and lighter to heavier wine styles [14]. As such, the more complex wines made via traditional production methods are not necessarily the preferred sparkling wine style [3,7]. Production process information can impact consumer expectations of quality and liking, but does not always affect informed liking [15]. Consumer trials suggest the varietal composition [16], and levels of carbon dioxide (effervescence) and dosage (sweetness) [17,18], can also influence tasting thresholds and sparkling wine preferences. However, it should be noted that the timing of consumption (relative to pouring) [19] and nucleation sites present in sparkling wine glasses [20] can significantly impact the organoleptic perceptions of carbon dioxide, i.e. the appearance, taste and texture of bubbles or ‘fizz’. 

Extrinsic cues, including the country or region of origin, brand, recommendations, price, occasion and symbolism, are also important drivers of the perceived quality of sparkling wine [7,21,22,23], and thus, influence sparkling wine purchasing decisions [23]. However, the relative importance of these drivers can vary amongst wine consumers from different countries. For example, consumers from the United Kingdom value traditional advertising that focuses on the product itself, whereas Australian, New Zealand and US consumers tend to focus more on the image, and the enjoyment and fun associated with sparkling wine consumption [24]. Similarly, sparkling wine consumption in Croatia is often associated with specific celebrations [25]. Consumers are usually willing to spend more on sparkling wine purchased for special occasions [26,27], demonstrating the importance of situational context. Country of origin and price have been shown to influence consumer perception of prestige and luxury [26], albeit in a more recent study, Australian sparkling wine consumers denied kudos (i.e., prestige/status) motivated their Champagne purchases [28]. Price continues to be a strong driver of wine purchasing decisions [8,9,10,29,30,31,32,33], and consumers often associate higher prices with superior quality [29,30].

Segmentation is often performed to study the preferences of specific groups of wine consumers. For example, extensive research has been published concerning generation Y consumers’ attitudes towards, and preferences for, Champagne and sparkling wine [34,35,36,37,38,39]. Gender is also thought to influence the frequency of sparkling wine consumption. Whereas Lerro and colleagues reported similar rates of sparkling wine consumption by men and women in the US [40], other studies suggest the volume [41] and type/style [42,43] of wine consumed, as well as occasions at which wine is consumed [44], are all influenced by gender. Wine involvement is also considered to play an important role in determining consumer preferences and behavior [3,27]. The Fine Wine Instrument (FWI) is a statistical tool developed to segment consumers based on wine connoisseur, knowledge and provenance variables [45]. The FWI classifies consumers as ‘No Frills’, ‘Aspirants’ and ‘Enthusiasts’ and is an appropriate model for segmenting sparkling wine consumers, given sparkling wines are often categorized as luxurious products [46].

This study examined the influence of fine wine knowledge and behavior (determined using the FWI) on consumer perceptions of and preferences for different styles of sparkling wine. Consumers’ familiarity with and ability to identify different sparkling wine styles were also explored, as well as knowledge of sparkling white wine production methods. In this way, the study aimed to provide insight into consumers’ expectations of sparkling wine and the importance of the consumption context. The results from this work will enable industry to tailor their marketing strategies for different sparkling wine styles to specific segments of the domestic market. Research in the field of wine science is also advanced through a novel application of the FWI. 

## 2. Materials and Methods 

### 2.1. Sparkling Wines

Nine commercial wines, a French Champagne and eight Australian sparkling wines (Table 1), were chosen in consultation with an industry reference group comprising four prominent Australian sparkling winemakers. The Australian sparkling wines included four sparkling white wines made via carbonation, Charmat, transfer and Méthode Traditionelle production methods (hereafter CA, CH, TR and MT, respectively), a sparkling red wine, a sparkling rosé wine, a Moscato and a Prosecco. A French Champagne was included, to reflect the international benchmark for sparkling wine. Wines were chosen to be representative of each wine style (i.e., to reflect sensory profiles typical of each style, as well as prominent brands in the domestic market); in the case of the sparkling white wines and Moscato, quality ratings and wine sensory profiles available from two previous studies [3,4] were used to inform wine selection. Wines were then sourced from retail outlets and cellared at 15 °C until required.

### 2.2. Chemical Analysis of Wines

Aliquots of sparkling wine (~50 mL, taken from three separate bottles of each wine) were degassed using an ultrasonic bath (Sonorex Digitec DT 1028F, Bandelin Electronic, Berlin, Germany) as described previously [47]. The basic composition of degassed wines were then determined using published analytical methods [48]. pH and titratable acidity (TA, expressed as g/L of tartaric acid) were measured with an autotitrator (Compact Titrator, Crison Instruments, Allela, Spain). Ethanol (as percentage alcohol by volume, abv) was measured with an alcolyzer (Anton Paar, Graz, Austria). Residual sugar was measured enzymatically with a d-glucose/d-fructose enzymatic test kit (Boehringer-Mannheim, R-BioPharm, Darmstadt, Germany), using a liquid handling robot (CAS-3800, Corbett Robotics, Eight Mile Plain, Qld., Australia) and a spectrophotometric plate reader (Infinite M200 Pro, Tecan, Grödig, Austria). Wine color was determined via spectral (CIELAB) measurements performed with a Cintra 4040 spectrometer (GBC Scientific Equipment, Melbourne, Vic., Australia), operating between 380 and 780 nm (at 2 nm intervals). Total phenolics were measured as the absorbance of wine at 280 nm using the Cintra 4040 spectrophotometer. 

### 2.3. Descriptive Analysis of Wines

The sensory profiles of sparkling wines were determined by descriptive analysis (DA) [49] with a trained panel of 12 judges (10 females and 2 males, aged between 18 and 50 years) comprising University of Adelaide staff and students. Panelists were recruited on the basis of their availability and previous wine sensory experience (including DA of sparkling white wines [3] and/or Moscato [4]). The panel completed eight hours of training (4 × 2 h sessions over four weeks) during which they identified descriptive terms and gained familiarity in recognizing and scoring the intensity of each attribute [50]. DA training also included practice evaluation sessions, conducted in isolated sensory booths under the conditions used during formal assessment (i.e., controlled ventilation, red lighting and a temperature of 22–23 °C). This also enabled evaluation of panel performance (reproducibility and repeatability). The panel generated 38 attributes, including: apple/pear, bruised apple, citrus, confectionary, dark fruit, floral/musk, honey, mixed spice, oaky, savory/smoky, stone fruit, toasty/nutty, tropical fruit, vanilla/caramel and yeasty aromas and flavors; overall aroma and flavor intensity; and sweetness, bitterness, acidity, astringency, complexity and effervescence. Reference standards were developed (Table S1) and provided at subsequent training sessions and during final evaluations, and panelists could refer to these at any time during evaluations. 

Throughout DA sessions (both training and formal evaluation), a standardized protocol was employed to minimize variability in pouring and serving wines, and changes in wine carbonation and temperature [3,19]. Wines were poured immediately prior to evaluation, with glasses held at a 45° angle and wine (~30 mL) poured down the inside of the glass. Wines were served chilled (i.e., at 5 °C), in air-dried, three digit-coded black XL5 (ISO standard) 215 mL stemmed wine glasses (covered with lids). Panelists received wines from the same bottles, which were sealed with sparkling wine stoppers and refrigerated between pours. 

Three formal evaluation sessions were held, with 9 wines presented in each session, such that all wines were assessed in triplicate. Wines were presented in a randomized order (across panelists), in brackets of three to minimize warming and loss of carbon dioxide. The time lapse between pouring and serving wines was less than 30 sec, with panelists completing evaluation of brackets in five to eight min. Breaks (3 min) were enforced between each bracket to avoid sensory fatigue. Distilled water and plain crackers were provided as palate cleansers. Panelists rated the intensity of each sensory attribute using 15 cm unstructured line scales, with anchor points of ‘low’ and ‘high’ placed at 10, and 90% on the scale, respectively. Data were acquired with FIZZ software (Version 2.47b, Biosystèms, Couternon, France).

### 2.4. Consumer Trials

Consumer trials were completed within 1 month of DA. Regular sparkling wine consumers (*n* = 203) were recruited using various methods, including flyers, e-newsletters, social media and an internal wine consumer database. Inclusion criteria required participants to be at least 18 years of age and regular consumers of sparkling wine (i.e., ≥12 times per year). Consumers attended a single tasting session, during which they rated their acceptance of a subset of the sparkling wines, but in the fortnight prior to the consumer tasting, they first completed an online survey. 

#### 2.4.1. Online Survey

The online survey, administered via SurveyMonkey™ (San Mateo, CA, USA), was adapted from a previous study [27] and took participants 10–15 min to complete. The first section of the survey comprised demographic questions related to sex, age, education, household income and alcohol consumption. The second section then explored participants’ knowledge of, and preferences for, different styles of sparkling wine. Participants were made aware that sparkling wine should only be called Champagne if it comes from the region of Champagne in France, but that for the purposes of this study, all other sparkling wine styles should be assumed to be Australian in origin. Participants were asked to: (i) list words they associated with each style of sparkling wine; (ii) indicate their liking of each style of sparkling wine (using 9-point category scales, where 1 = extremely dislike, 5 = neither like nor dislike and 9 = extremely like); and (iii) indicate how frequently they consume each style of sparkling wine at a number of pre-determined occasions (using 9-point category scales, where 1 = never, 5 = sometimes and 9 = always). Participants were also asked to rate their familiarity with different sparkling wine production methods (carbonation, Charmat, transfer and Méthode Traditionelle; again using 9-point category scales) and the price they would typically spend (in Australian dollars) for a 750 mL bottle of each style of sparkling wine at a retail outlet (response options were: never purchase; <$15; $15–$29; $30–$49; $50–$79; and >$80). 

#### 2.4.2. Acceptance Testing

Consumer acceptance testing was undertaken over a four week period, in sensory laboratories at either the University of Adelaide’s Waite Campus or the University of South Australia’s City East Campus, under the same conditions used during DA (i.e. controlled ventilation, red lighting and a temperature of 22–23 °C). Prior to wine evaluation, participants completed the Fine Wine Instrument (FWI) survey, a statistical model developed to segment consumers (as ‘No Frills’, ‘Aspirants’ and ‘Enthusiasts’) according to their fine wine behavior and knowledge [45]. Participants were then instructed on how to assess wines using a hedonic scale, before being presented with wines. However, for ethical reasons, each consumer evaluated a subset of six sparkling wines (chosen randomly according to an incomplete block design). The standardized protocol described above for DA was again employed. Wines were presented in a randomized order, in brackets of three to minimize warming and loss of carbon dioxide. The time lapse between pouring and serving wines was less than 30 sec, with participants completing evaluation of brackets in five min. Participants rated their liking of each wine using 9-point hedonic scales (where 1 = extremely dislike, 5 = neither dislike nor like and 9 = extremely like). Consumers were also asked to identify how much they would expect to pay for a 750 mL bottle of each wine and the style of each sparkling wine. Data were acquired with Survey Monkey^TM^. On completion of wine evaluation, participants received a $20 gift voucher as compensation for their time. 

### 2.5. Data Analysis 

Statistical analyses were performed with XLSTAT 2012.1.01 (Addinsoft, New York, NY, USA). Chemical and DA data were analyzed by analysis of variance (ANOVA), with principal component analysis (PCA) of DA data also performed. Consumer data were analyzed using a combination of descriptive techniques (frequencies, percentages, medians and quartiles), agglomerative hierarchical clustering and non-parametric testing. Mood’s median test was used to test the equality of medians from two or more populations because the data was ordinal and did not follow normal distribution. Additionally, sample distribution shapes were different and variability was not constant across datasets. To compare proportions of consumers within each segment, a chi-square test and Marascuilo procedure was used. Fisher exact tests were also used to test the association between qualitative variables, given that some counts within contingency tables were less than 5, while a Wilcoxon signed-rank test compared median expected liking scores vs. median actual liking scores. Qualitative analysis of word frequencies was carried out with NVivo qualitative data analysis software (Version 10, QSR International Pty Ltd., Melbourne, Victoria, Australia). 

### 2.6. Ethical Statement

DA panelists and consumers gave informed consent before participating in the study, which was approved by the Human Research Ethics Committees of the University of Adelaide (Project No. H-212-2014) and the University of South Australia (00000338180).

## 3. Results and Discussion

### 3.1. Chemical and Sensory Profiles of Sparkling Wines

**Figure 1:**
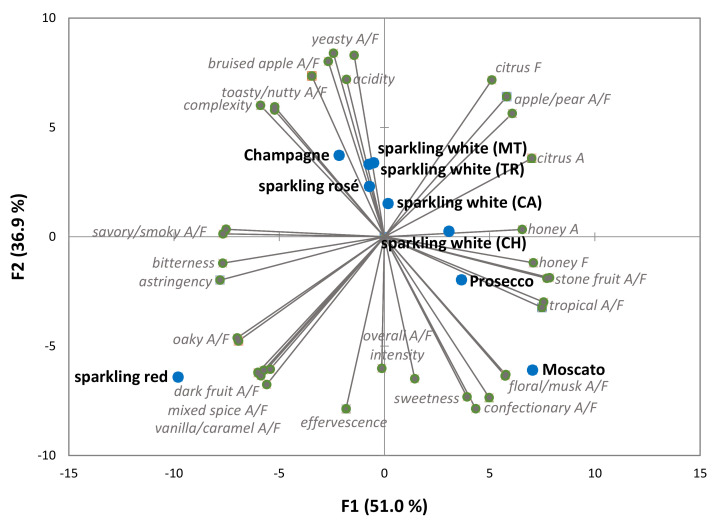
Principal component analysis biplot of sensory attribute ratings of the French Champagne and Australian sparkling wines studied. CA = carbonated; CH = Charmat; TR = Transfer; MT = Méthode Traditionelle; A = aroma; F = flavor.

Chemical and sensory analyses were performed prior to consumer trials to establish the compositional and sensory variation amongst the different sparkling wines (Table 2, Figure 1, Table S1). The wines were generally characterized by low pH (typically 3.00–3.12, albeit the pH of the sparkling rosé was 3.35), high TA (8.0–10.4 g/L), low residual sugar (<13 g/L), moderate alcohol (10.8–13.4% abv) and low phenolics (1.2–3.6 au), as would be expected of sparkling wine. The exceptions to this were: Moscato, for which the residual sugar was 56.5 g/L and alcohol content was 6.2% abv, which were consistent with previously reported compositional data for this sweeter, lighter-bodied style of sparkling wine [4]; and the sparkling red wine, which had considerably higher phenolics (51.2 au) as a consequence of alcoholic fermentation on skins and 23.0 g/L of residual sugar.

The sensory profiles of the various sparkling wines were determined by DA and significant differences were observed amongst the intensity ratings of all sensory attributes (Table S2), reflecting the stylistic diversity of the nine wines. PCA of sensory data gave the biplot shown in Figure 1 and the first and second principal components explained 51 and 37% of variation, respectively. The Champagne, sparkling white wines made via carbonation, transfer and Méthode Traditionelle production methods and sparkling rosé, were clustered around the middle of the two upper quadrants, based on the prominence of yeasty, toasty/nutty and bruised apple aromas and flavors, complexity and acidity. In contrast, the more fruit driven styles of sparkling wine, i.e., the Charmat, Prosecco and Moscato wines, were positioned in the quadrants on the right; with the Moscato strongly associated with floral/musk and confectionary attributes and sweetness, typical of this style. The sparkling red wine was situated in the lower quadrant on the left, reflecting the intensity of dark fruit, mixed spice, vanilla/caramel and oaky aromas and flavors. These results suggest sensory differences between the sparkling white (CH), sparkling red, Prosecco and Moscato wines should be more apparent than amongst the Champagne, sparkling white (especially the CA, TR and MT wines) and sparkling rosé wines. 

### 3.2. Consumer Perceptions of and Preferences for Different Sparkling Wine Styles

Two hundred and three consumers were recruited to participate in consumer trials (Table 3). A higher proportion of female consumers participated (61.5%), which might reflect gender-based preferences for sparkling wine [23,40], but this was consistent with demographics reported in other recently published sparkling wine studies [3,4,7,23,27]. Although all age categories were represented, 47.3% of participants were aged ≥55 years, and thus, older consumers were over-represented relative to younger consumers (only 16.7% of participants were aged <35 years). Whereas all participants consumed sparkling wine at least once per month (regular consumption of sparkling wine was one of the inclusion criteria for participation in the consumer trial), 25.1% of participants consumed sparkling wine one or more times per week, and a further 33.0% of participants consumed sparkling wine fortnightly (Table 3).

Hierarchical clustering based on responses to questions from the Fine Wine Instrument [45] was used to classify participants as ‘No Frills’ (*n* = 31), ‘Aspirant’ (*n* = 104) or ‘Enthusiast’ (*n* = 68) wine consumers (Table 3). No Frills consumers typically show little connoisseur-type behavior and have limited knowledge of wine or interest in wine provenance. They typically purchase their wine from chain retailers, rather than independent or fine wine retailers [45]. Aspirant consumers share some of the characteristics of Enthusiast consumers segment, but are not as knowledgeable, nor as confident or adventurous in their wine-purchasing abilities. Their purchases are predominantly from chain retailers and they are influenced by the others’ opinions (e.g., friends and family, staff at restaurants, wine retailers and wine writers), as well as advertising, promotions, and awards or medals [45]. In contrast, enthusiast consumers are knowledgeable about wine and actively enjoy increasing their knowledge. They exhibit connoisseur-like behavior (i.e., they tend to keep records of their wine purchases, have dedicated wine storage space and ritually check their wines for faults prior to consumption), purchase wine from independent wine retailers, and are adventurous in their wine purchasing (i.e., they like to try different wines). Enthusiasts are confident in their ability to select wines, but will also ask questions and/or seek recommendations [45]. In the current study, a significant proportion of No Frills consumers were female (i.e., 83.9%), whereas female consumers represented 58.7% and 55.9% of the Aspirant, and Enthusiast segments, respectively. The age, household income and level of education of the FWI segments were similar, but interestingly, the No Frills segment tended to consume sparkling wine more frequently (35.5% consumed sparkling wine at least once or more per week) and the Aspirant segment less frequently (81.7% consumed sparkling wine only fortnightly or monthly). 

On average, participants’ alcohol consumption predominantly comprised wine (at 68.2% of total alcohol consumption), with similar rates of wine consumption for each FWI segment (Table S3). This largely comprised red wine (42.4%), white wine (28.6%) and sparkling wine (23.2%) consumption, of which No Frills consumers consumed significantly more sparkling wine than Aspirant (*p* = 0.006) and Enthusiast (*p* < 0.001) consumers, while Enthusiasts consumed significantly more fortified wine than Aspirants (*p* = 0.030). When consumers’ sparkling wine consumption was considered, sparkling white wine (53.8%), sparkling red wine (22.4%) and Champagne (14.5%) accounted for >90% of total consumption (Table S3). This likely reflects the predominance of sparkling white wine in the domestic market [2]. Statistical analysis confirmed significantly higher consumption of sparkling white wine by No Frills consumers compared with Enthusiasts (*p* = 0.039), and higher consumption of sparkling red wine, but lower consumption of Prosecco by Aspirants compared with Enthusiasts (*p* = 0.012 and *p* = 0.017, respectively). Other potential differences in red and sparkling red wine consumption by No Frills consumers (relative to Aspirants and Enthusiasts) were not validated by statistical analysis, which was attributed to the comparatively small number of No Frills consumers (*n* = 31). 

Frequency analysis of the words consumers associated with different styles of sparkling wine provided insight into their perceptions of each wine style (Table 4). All styles were described as ‘bubbly’ (or ‘bubbles’ in the case of sparkling red wine), and Champagne, sparkling white wine and sparkling rosé wine were associated with celebration. The reputation of Champagne was evident from its association with ‘expensive’, ‘special’, ‘luxury’, ‘refined’ and ‘fine’, and it’s origin with reference to the word ‘French’. Sparkling white, rosé and Moscato wines were described as ‘light’ ‘refreshing’ and ‘fun’, whereas sparkling red was considered to be ‘rich, ‘dark’ and ‘heavy’. The most frequently used word, ‘sweet’, was offered by 184 participants to describe Moscato, demonstrating consumers’ familiarity with the characteristic sweetness of this style of sparkling wine; albeit, the use of the word ‘sickly’ would suggest the style does not appeal to all consumers. In contrast, consumers were clearly less familiar with Prosecco. Almost 20% of participants described Prosecco as ‘sweet’, whereas typically it is a dry style of sparkling wine, while ‘don’t know’, ‘none’ and ‘sounds familiar’ were all amongst the more frequently used descriptors. Nevertheless, the reference to ‘Italy’ and ‘Italian’ indicates consumer awareness of the geographical origin of Prosecco. These findings were consistent with results from an online survey of Australian sparkling wine consumers [27].

During blind tastings, consumers rated their liking of a randomly chosen subset of six of the nine sparkling wines (Table 5). Mean liking scores ranged from 4.8 for Moscato to 6.0 for the carbonated sparkling white wine; these wines also received the lowest (5.0) and highest (7.0), median liking scores, respectively. This was consistent with two previous studies which found on average, Australian consumers liked fruit-driven Charmat sparkling white wines more than more complex transfer and Méthode Traditionelle sparkling wines [3,7], and in one of these studies, Champagne [7].

The interquartile ranges (IQR = 3rd quartile–1st quartile) for the liking scores of each sparkling wine ranged from 2 to 6 for No Frills and Enthusiast consumers, and by 2 to 5 for Aspirants, indicating that there was considerable variation amongst consumer liking scores, even within FWI segments. Nevertheless, statistically significant differences in liking were observed. No Frills consumers tended to like the CH and MT sparkling white wines and Champagne more, and the sparkling rosé wine and Moscato less, whereas Aspirants liked the sparkling rosé wine and the CH and TR sparkling white wines. Enthusiasts liked the sparkling red wine the most, followed by the Champagne, the CA sparkling white and sparkling rosé wines, and they liked the Moscato the least. Statistical analysis revealed the No Frills consumers’ liking scores for the CH and MT sparkling white wines were significantly higher than the corresponding scores of Aspirants, but Aspirant consumers liking scores for sparkling rosé wine were significantly higher than for No Frills consumers. Aspirants’ liking of Moscato was neutral (5.3), but it was significantly higher than for Enthusiasts (4.4). Despite consumers’ limited familiarity with Prosecco (Table 4), mean liking scores for Prosecco were neutral to favorable (i.e., 5.2–5.9), irrespective of FWI segment. Whereas the Champagne, sparkling white (TR and MT especially) and sparkling rosé wines exhibited varying levels of complexity, together with underlying apple/pear, citrus and stone fruit aromas and flavors, and crisp acidity, the Prosecco displayed intense fruity (apple/pear, citrus, tropical, stone fruit), floral and confectionary characters, and less acidity (Figure 1, Table S2), which some consumers might have found more amenable. The apparent sweetness of the Moscato (Figure 1) clearly doesn’t appeal to all consumers, while the red fruit and oak notes (Figure 1) and fuller body (Table 2) exhibited by the sparkling red tended to appeal to Aspirants and Enthusiasts, who typically drink more red wine (Table S3).

Previous research involving segmentation of consumers based on their liking of different sparkling white wines found younger consumers tended to prefer more fruit-driven sparkling wine styles (i.e., CA and CH sparkling wines), while older consumers appreciated the more complex TR and MT sparkling wines [3]. The authors hypothesized this might in part reflect the demographics of consumer segments; i.e., older consumers, particularly those with higher household incomes, can afford to consume higher priced sparkling wines more frequently than younger, less affluent consumers. In the current study, the Enthusiast segment did comprise a higher proportion of more affluent consumers (than other FWI segments), but Enthusiasts tended to like a broad range of wines, including the CA sparkling white wine. 

### 3.3. Consumer Knowledge of Sparkling Wine Production Methods

Disclosure of country of origin and method of production provide extrinsic cues which can influence consumers’ perception of sparkling wine quality and/or hedonic liking [3,7,15,25]. Consumers were therefore asked to rate their knowledge of Champagne and sparkling white wine production methods, from 1 = extremely unfamiliar to 9 = extremely familiar (Table 6). Mean responses ranged from 1.9 for Charmat production to 3.6 for Méthode Champenoise, indicating consumers had limited appreciation of sparkling winemaking. Irrespective of the FWI segment, higher responses were given for Méthode Champenoise, carbonation and Méthode Traditionelle (than the Charmat and Transfer methods), with responses given by the more knowledgeable Aspirant and Enthusiast consumers being significantly higher than responses from No Frills consumers. The IQRs for responses from No Frills, Aspirant and Enthusiast consumers were 0–1, 1–5 and 2–6, respectively, which suggests some Aspirants and Enthusiasts were familiar with some production methods. Enthusiasts knowledge of the traditional Méthode Champenoise and Méthode Traditionelle was significantly higher than that of Aspirants (*p* = 0.025, and *p* = 0.001, respectively). However, since consumers were not specifically asked to explain their understanding of the different production methods, it is not clear to what extent consumer responses reflect awareness that there are different methods versus a true appreciation of what each method involves and how this influences wine sensory properties and quality. Given that most consumers had limited knowledge of sparkling winemaking, there were low expectations of consumers’ ability to identify the different styles of sparkling wine presented during the blind tasting, with the exception of the sparkling red and Moscato wines, which both exhibited distinctive sensory profiles; dark fruit and oak aromas and flavors in the case of the sparkling red wine, and varietal fruit, floral, musk and confectionary characters and apparent sweetness in the case of the Moscato (Figure 1).

### 3.4. Consumer Recognition of Different Sparkling Wine Styles

As expected, of the nine different sparkling wines evaluated in the current study, only the Moscato and sparkling red wines were identifiable by a majority of consumers, i.e. by 79.6% and 59.2% of consumers, respectively (Table 7). Presumably the characteristic confectionary and floral/musk notes, and sweetness of Moscato, and the dark fruit and oak aromas and flavors exhibited by the sparkling red wine (Figure 1, Table S1), contributed to the relative ease of their identification.

Surprisingly, a substantial proportion of consumers (~17–27%), including Enthusiasts, were unable to identify the Moscato. The use of black wine tasting glasses (which concealed color) confounded identification of the sparkling red wine for ~40% of consumers, despite its distinct sensory profile (Figure 1). This highlights the difficulty consumers, even knowledgeable consumers, have evaluating wine, especially without visual or extrinsic cues (such as brand, region, variety and/or price).Only 19% of consumers correctly identified the Champagne, with a small, but significantly higher proportion of No Frills consumers identifying the Champagne than Enthusiasts (*p* < 0.003). However, given the limited number of No Frills consumers (*n* = 31), this result might not be representative. Fewer than 10% of consumers were able to identify the sparkling white, rosé and Prosecco wines, which likely reflects both the similarity amongst the sensory profiles of these wines (Figure 1) and consumers’ limited knowledge of sparkling winemaking methods. Despite the bottled fermented sparkling white wines (i.e., TR and MT sparkling white wines) exhibiting toasty, yeasty notes that were not evident in the CA and CH sparkling white wines or the Prosecco, the majority of consumers were not able to distinguish the different sparkling white wine styles. Indeed, 131/203 consumers gave responses of ‘Unsure’ for one or more of the Australian sparkling white wines (data not shown). The low identification rate observed for Prosecco (5.8%), again suggests consumers are not familiar with this wine style.

### 3.5. Comparison of Consumer Expected vs. Actual Liking of Different Sparkling Wine Styles

In the online survey (i.e., prior to the blind tasting), consumers were asked to rate their expected liking of the different styles of sparkling wine (Table 8). Responses indicated consumers expected they would like Champagne the most (9.0), followed by sparkling white wine (8.0), sparkling red wine (7.0) and sparkling rosé wine (6.0); neutral scores (i.e., 5.0) were given to Moscato and Prosecco. 

After the blind tasting, a Wilcoxon signed-rank test compared median expected liking scores with median actual liking scores, and found consumers’ expected liking of Champagne and sparkling white red wines was significantly higher than the actual liking scores awarded to these wines during the tasting (*p* < 0.0001). It is possible that the clinical nature of the sensory laboratory (i.e., the individual white booths, red lighting, and opaque wine glasses), as compared with the contextual settings typically experienced during wine consumption, resulted in the (untrained) consumers being hyper-analytical of the wines they evaluated. However, the differences in liking scores might also be explained by consumers being unable to differentiate wine styles and/or wine quality, in the absence of extrinsic cues. Thus, during the tasting, more conservative (neutral) liking scores were given. A third explanation might be that the nine sparkling wines studied, didn’t match consumers’ expectations of the different sparkling wine styles.

Aspirants and Enthusiasts significantly over-estimated their liking of Champagne and sparkling white and red wines. In the case of Champagne, the tradition, heritage and prestige associated with French Champagne likely influenced consumers’ perceptions of quality, and therefore, expected liking, as reported in previous studies [7,21]. No Frills consumers predicted their moderate liking of Champagne, but over-estimated their liking of sparkling white wine, and Moscato in particular. Interestingly, Enthusiasts anticipated liking Moscato the least, and this was reflected in the liking scores given to the Moscato during the tasting, which, despite wines being presented blind, the majority of Enthusiasts (i.e., 72.7%, Table 8) correctly identified. The expected and actual liking scores for Prosecco were equal to, or higher than for Moscato, suggesting Prosecco might fare well in the Australian domestic market, once consumers gain greater familiarity of this style of sparkling wine. This represents an opportunity for Australian producers of Prosecco to consider how they market this style of wine to their consumers, especially Aspirants and Enthusiasts.

### 3.6. Sparkling Wine Consumers’ Purchasing Behavior

The bottle price and quality:price ratio were found to be amongst the most important sparkling wine characteristics driving the purchasing decisions of Croatian sparkling wine consumers [25], and may similarly play a role in Australian sparkling wine consumers’ purchasing decisions. In the current study, consumers rarely spend more than $50 (AUD) per bottle for Australian sparkling wine and only 47 consumers (comprising 36% of Aspirants and 37% of Enthusiasts) typically spend $50 or more on Champagne (Table 9). A substantial proportion of consumers (from all FWI segments) indicated they never purchase sparkling rosé (51/139, 36.7%), sparkling red (31/130, 23.8%), Moscato (66/137, 48.2%) or Prosecco (93, 67.4%) wines. These results suggest consumers either do not like or do not expect to like these sparkling wine styles, in agreement with consumers’ expected liking scores (Table 8). In contrast, only two consumers indicated they never purchase sparkling white wine, consistent with consumers’ self-reported sparkling wine consumption (Table 4). 

Of those consumers who purchase the various styles of Australian sparkling wine, the majority (~73–97%) spend less than $30 per bottle (Table 9); only 16% and 27% of consumers indicated they spend more than $30 per bottle for sparkling white and red wines, respectively. As expected, a higher proportion of the consumers who tend to spend more on the different styles of sparkling wine were Aspirants and Enthusiast, albeit the typical spend by FWI segment was only statistically significant for Champagne (*p* = 0.005) and Prosecco (*p* < 0.0001). To some extent, these results reflect the bottle prices for the different sparkling wine styles; certainly the Moscato and Prosecco sourced for the current study had lower retail prices than the sparkling rosé and red wines, which were in turn lower in price than the Méthode Traditionelle wine and the Champagne (Table 1). Of course, as with all wine styles, there can be considerable price variation even amongst sparkling wines of the same style.

The blind tasting results again demonstrated the difficulty consumers had identifying sparkling wine styles and their preconceptions of the sensory profiles of different styles of sparkling wine. Whereas 67% of consumers (93/138) indicated they never purchase Prosecco in their survey responses, only a quarter gave this response in the tasting (Table 7), which reflects the consumers’ inability to recognize this sparkling wine style. In the case of Moscato, the most readily identifiable sparkling wine, 46% of consumers (63/137) indicated they never purchase Moscato based on their tasting (compared with 48% in the survey). Consumers seemingly misidentified the Prosecco, which might explain why the expected liking scores and typical spend for Prosecco were low (Table 8 and Table 9), yet 12% of consumers who tasted the Prosecco (i.e., 17/138) indicated they would spend more than $30 per bottle for this wine (Table 7). Previous research has shown that in Italy, consumers use price as an indicator of Prosecco quality [51] and that higher prices can instill high consumer loyalty [52]. In Australia, this may be true of some sparkling white wines, but would not be expected to be the case for Prosecco until consumer familiarity with this sparkling wine style improves.

The results from the tasting indicated the typical spend by FWI segment was only statistically significant for sparkling rosé wines (*p* = 0.048), with a higher proportion of Aspirants and Enthusiasts again indicating they would spend more per bottle than No Frills consumers (Table 9). The association between survey and tasting scores was also compared by FWI segment using Fisher Exact tests. Significant differences were only observed between responses from Aspirants (*p* = 0.014) and Enthusiasts (*p* = 0.017) for Moscato (Table 9), suggesting these consumers might spend more per bottle for Moscato based on their tasting experience, compared to their preconceived perceptions of this sparkling wine style. This finding was consistent with a recent study that found broad appeal for Australian Moscato wines [4].

### 3.7. Sparkling Wine Consumers’ Consumption Behavior

Numerous studies have shown that consumers associate sparkling wine with celebration and that they therefore tend to consume sparkling wine at special occasions [25,26,27,30,53,54,55]. In the current study, consumers similarly indicated they consumed Champagne and sparkling white wine at anniversaries, birthdays, Christmas, New Year and weddings, with sparkling white wine also consumed at the Melbourne Cup and at restaurants and cafes (Table S4). Aspirants and Enthusiasts also consumed sparkling red wine at Christmas, whereas median responses of 1.0–1.5 indicated Moscato and Prosecco were not styles of sparkling wine that were consumed very often at any of the suggested occasions or contexts. Significant differences were observed amongst some FWI segments’ survey responses (Table S5), but in most instances median responses were ≤2.0, so differences were not considered meaningful. Some meaningful differences were observed amongst FWI segments’ responses during the blind tasting (Tables S4 and S5). No Frills consumers were more likely to consume the Champagne at a girls’ or boys’ night out than Aspirants or Enthusiasts, and the MT sparkling white wine to celebrate a New Year, wedding, or at a restaurant or café, on weekends or at work drinks than Enthusiasts. However, they were less likely to consume the sparkling rosé than Aspirants and/or Enthusiasts to celebrate an anniversary, birthday, New Year or wedding.

Whereas survey responses indicated Champagne and sparkling white wine were the most frequently consumed sparkling wine at key celebratory occasions (i.e., anniversaries, birthdays, Christmas, New Year and weddings), with the exception of the Champagne at birthdays, these sparkling wine were given significantly lower ratings during the blind tasting (Table S4 and Table 10).

In the case of the sparkling rosé, Aspirant and Enthusiast scores were significantly higher (than survey responses) for all occasions and contexts, suggesting these FWI segments liked this wine (Table 8) and could envisage consuming it, particularly at celebratory occasions. Enthusiasts also gave significantly more favorable responses for consumption of the sparkling red wine at birthdays, New Year, weddings, restaurants and cafes, and on weekends. Interestingly, Moscato ratings increased for all occasions and contexts, often irrespective of FWI segment, but not to the same levels as the other sparkling wine styles. Lastly, all three FWI segments indicated they would be more likely to consume the Prosecco, regardless of occasion or context, than previously suggested by their survey responses. Once again, this was consistent with the observed increased in expected vs. actual liking scores for (Table 8), as well as the higher spend price per bottle (Table 9), for this wine. However, given that consumers were not familiar with this sparkling wine style (Table 6) and could not differentiate it from other sparkling wines (Table 7), this might again be attributed to consumers believing the Prosecco to be a different wine, e.g., the Champagne or MT sparkling white wine.

## 4. Conclusions

Consumers tended to associate sparkling wine with celebration, but it was clear that the various sparkling wine styles were perceived differently. Champagne was recognized as an expensive, but refined, luxury product, while sparkling white and rosé wines were described as light and refreshing. However, consumers could not readily differentiate sparkling white wine styles, likely due to their limited knowledge of sparkling wine production methods. In contrast, ~80% of consumers correctly identified the Moscato based on its prominent fruit character and sweetness, and 60% of consumers identified the sparkling red wine, which exhibited dark fruit and oak aromas and flavors. Consumers were far less familiar with Prosecco; indeed two thirds of consumers indicated they never purchase Prosecco.

Consumers anticipated liking Champagne and sparkling white wine the most, and Moscato and Prosecco the least, however upon tasting, significantly lower liking scores were given to the Champagne, and sparkling white and red wines. Liking scores for the fruit driven Moscato and Prosecco wines were comparable with their expected liking scores, but surprisingly, No Frills consumers liked these two sparkling wine styles the least. This might, however, reflect the limited size of this FWI segment (*n* = 31), which is attributed to self-selection biases and acknowledged as a limitation of the current study. Nevertheless, despite two thirds of consumers indicating they never purchased Prosecco, this sparkling wine style was rated favorably, and consumers from all FWI segments could envisage consumption of Prosecco at a range of occasions. Similarly, while sparkling rosé only accounted for ~10% of participants’ total sparkling wine consumption, consumers (especially Aspirants) liked this wine style, and on tasting, Aspirants and Enthusiasts indicated they would likely consume sparkling rosé at similar occasions as for the Champagne. This research suggests there may be opportunities for wine marketers to better position these wine styles in the domestic market and to actively promote Prosecco to sparkling wine consumers, many of whom might currently avoid the style because it is not familiar, and thus, represents a purchasing risk. 

Consumers’ preconceived expectations of different sparkling wine styles clearly influences both their purchasing and consumption behavior, i.e., the price they are willing to pay per bottle, their expected liking and the occasions at which they would consume different styles of sparkling wine. Significantly higher expectations were held for Champagne and sparkling white wine, especially by Aspirants and Enthusiasts, who were more likely to spend more per bottle for these styles of sparkling wine. Consumption of these sparkling wines was strongly associated with celebratory events such as anniversaries, birthdays, Christmas, New Year and weddings; sparkling red wine consumption was most frequently associated with Christmas. However, without extrinsic cues, consumer perceptions of Champagne and sparkling white and red wines seemingly decreased, whereas, perceptions of sparkling rosé and Prosecco were similar, or improved.

This study advances our understanding of the factors that influence sparkling wine consumers’ purchasing and consumption behavior, i.e., not just age and gender, but the wine connoisseur, knowledge and provenance variables that underpin the FWI. Collectively, the results provide sparkling wine producers important insight into consumers’ perceptions, expectations and perceptions of different styles of sparkling wine, i.e., information which can be used to tailor marketing strategies for specific sparkling wine styles and/or towards specific consumer segments. For example, strategies that introduce consumers to lesser known styles, such as Prosecco, to build familiarity, or that showcase style variation to breakdown negative pre-conceptions/expectations or enhance the quality:price ratio of bottle-fermented sparkling white wines. The current study focused on Australian consumers and Australian sparkling wines, with Champagne included as the international benchmark for sparkling wine, nevertheless research findings are likely to be relevant to other New World sparkling wine markets. Furthermore, the study could be replicated elsewhere, to enable cross-cultural comparisons of sparkling wine purchasing and consumption behavior.

Finally, one additional limitation to the current study should be acknowledged: whilst the sparkling wines presented to consumers during acceptance testing were chosen to be representative of their respective wine styles, different results may have been obtained with a different selection of sparkling wines, due to style variation. 

## Figures and Tables

**Table 1:** Vintage, varietal composition, geographical origin and price of the French Champagne and Australian sparkling wines studied.

Wine Style	Vintage	Varieties	Region	Price (AUD)
Champagne	NV	PN, Ch, PM	Champagne	55
Sparkling white (CA)	NV	Ch, PN	SA	25
Sparkling white (CH)	NV	Ch, PN	SE Australia	10
Sparkling white (TR)	NV	PN, Ch, PM	SA, NSW, Vic.	30
Sparkling white (MT)	2008	PN, Ch	Vic.	40
Sparkling red	2012	Shiraz	Vic.	20
Sparkling rosé	NV	PN, Ch	Tas.	25
Moscato	2012	Muscat	Vic.	15
Prosecco	NV	Glera	Vic.	15

AUD = Australian dollars; CA = carbonated; CH = Charmat; TR = Transfer; MT = Méthode Traditionelle; NV = non-vintage; Ch = Chardonnay; PM = Pinot Meunier; PN = Pinot Noir; NSW = New South Wales; SA = South Australia; SE = South Eastern; Tas. = Tasmania; Vic. = Victoria.

**Table 2:** Basic chemistry of the French Champagne and Australia sparkling wines studied.

Sparkling Wine	pH	TA (g/L)	Residual Sugar (g/L)	Alcohol (% abv)	Phenolics (au)
Champagne	3.00 ^e^	8.89 ^c^	8.6 ^cd^	12.7 ^c^	1.3 ^d^
Sparkling white (CA)	3.04 ^d^	10.43 ^a^	8.9 ^cd^	10.8 ^h^	2.7 ^c^
Sparkling white (CH)	3.12 ^b^	8.79 ^cd^	12.8 ^c^	11.1 ^g^	3.6 ^b^
Sparkling white (TR)	3.00 ^e^	8.77 ^d^	9.6 ^cd^	11.5 ^f^	1.2 ^d^
Sparkling white (MT)	3.11 ^bc^	8.27 ^f^	7.0 ^d^	13.5 ^a^	1.2 ^d^
Sparkling red	3.35 ^a^	9.23 ^b^	23.0 ^b^	12.8 ^b^	51.2 ^a^
Sparkling rosé	3.04 ^d^	8.60 ^e^	9.0 ^cd^	12.0 ^d^	2.3 ^c^
Moscato	3.10 ^c^	7.99 ^g^	56.5 ^a^	6.2 ^i^	2.5 ^c^
Prosecco	3.09 ^c^	8.28 ^f^	11.5 ^cd^	11.7 ^e^	2.0 ^c^
ANOVA *p*-Value	<0.0001	<0.0001	<0.0001	<0.0001	<0.0001

Values are means of three replicates (*n* = 3). Different letters (within columns) indicate statistical significance (*p* ≤ 0.05, one-way ANOVA). Titratable acidity (TA) measured as g/L of tartaric acid. CA = carbonated; CH = Charmat; TR = Transfer; MT = Méthode Traditionelle.

**Table 3:** Demographics and sparkling wine consumption of consumers and of Fine Wine Instrument consumer segments. Data represent response number (frequency) and proportion (percentage).

		All Consumers (*n* = 203)		No Frills (*n* = 31)		Aspirants (*n* = 104)		Enthusiasts (*n* = 68)	
Gender	Female	125	61.5	26	83.9	61	58.7	38	55.9
	Male	78	38.5	5	16.1	43	41.3	30	44.1
Age (years)	18–24	8	3.9	4	5.9	3	2.9	1	3.2
	25–34	26	12.8	7	10.3	14	13.5	5	16.2
	35–44	34	16.8	8	11.7	22	21.2	4	12.9
	45–54	39	19.2	11	16.2	21	20.2	7	22.6
	55–64	67	33.0	25	36.8	30	28.9	12	38.7
	>65	29	14.3	13	19.1	14	13.5	2	6.5
Household income (AUD)	<50,000	44	21.6	16	23.5	21	20.2	7	22.6
	50,000–100,000	84	41.4	29	42.6	45	43.3	10	32.3
	100,001–150,000	46	22.7	14	20.6	23	22.1	9	29.0
	>150,000	29	14.3	9	13.2	15	14.4	5	16.1
Education	High school	38	18.7	15	22.1	16	15.4	7	22.6
	Trade	43	21.2	11	16.2	25	24.0	7	22.6
	Undergraduate	55	27.1	20	29.4	25	24.0	10	32.3
	Postgraduate	67	33.0	22	32.4	38	36.5	7	22.6
Sparkling wine consumption	Once per month	85	41.9	10	32.3	47	45.2	28	41.2
	Once per fortnight	67	33.0	10	32.3	38	36.5	19	27.9
	Once per week	41	20.2	7	22.6	18	17.3	16	23.5
	>Once per week	10	4.9	4	12.9	1	1.0	5	7.4

Gender was the only demographic for which responses were significant (*p* ≤ 0.05, Fisher’s Exact Test).

**Table 4:** Frequencies and weighted percentages of the top ten words (and their synonyms) that consumers associated with different sparkling wine styles.

Word | Frequency | Weighted Percentage								
**Champagne**			**Sparkling White**			**Sparkling Red**		
expensive	61	10.1	bubbly	67	11.0	rich	52	6.8
celebration	44	7.3	celebration	41	6.9	red	39	6.6
bubbly	36	6.0	refreshing	51	6.8	bubbles	35	6.0
special	21	3.4	light	40	4.5	dark	16	2.6
luxury	21	3.1	fun	26	4.3	heavy	17	2.6
refined	14	2.5	summer	15	2.5	delicious	15	2.6
fine	13	2.5	fresh	30	2.4	Christmas	13	2.2
dry	12	2.0	crisp	15	2.2	sweet	14	2.1
French	12	2.0	happy	13	2.2	bodied	12	2.0
sparkling	14	1.9	drink	12	1.9	wine	12	2.0
**Sparkling Rosé**			**Moscato**			**Prosecco**		
pink	39	8.7	sweet	184	40.2	sweet	39	9.2
sweet	42	8.6	light	24	4.2	Italian	30	7.5
light	46	8.2	sickly	17	3.8	don’t know *	21	5.2
bubbly	28	6.2	bubbly	17	3.5	sparkling	21	4.2
refreshing	18	3.4	fruity	11	2.5	wine	16	4.0
fun	12	2.7	refreshing	13	2.3	none *	14	3.5
red	11	2.4	drink	9	2.0	dry	12	3.0
nice	10	2.2	wine	9	2.0	Italy	12	3.0
wine	9	2.0	fun	8	1.8	bubbly	12	2.7
celebration	8	1.8	low alcohol	6	1.4	sounds familiar *	10	2.5

Descriptors which indicated consumers were not familiar with the sparkling wine style are marked with an asterisk; with ‘none’ interpreted as no words could be associated with the wine style. Consumers were asked to the list words that they associated with each sparkling wine style (as many or as few words as desired, but at least one response).

**Table 5:** Liking scores of consumers and Fine Wine Instrument consumer segments for the different sparkling wines.

**1st Percentage Quartile | Mean | Median | 3rd Percentage Quartile**																
	**All Consumers**				**No Frills**				**Aspirants**				**Enthusiast**			
Champagne	4.0	5.9	6.0	7.0	4.0	6.3	7.0	8.0	4.0	5.7	6.0	7.0	5.0	6.1	7.0	7.5
Sparkling white (CA)	5.0	6.0	7.0	7.0	5.0	5.9	6.0	7.0	5.0	6.0	7.0	7.0	5.0	6.1	7.0	8.0
Sparkling white (CH)	5.0	5.9	6.0	7.0	5.0	6.5	7.0	8.0	5.0	6.0	7.0	7.0	3.3	5.4	6.0	7.0
Sparkling white (TR)	4.0	5.6	6.0	7.0	3.5	5.5	5.0	7.5	4.0	5.5	6.0	7.0	5.0	5.9	6.0	7.0
Sparkling white (MT)	3.0	5.3	6.0	7.0	6.0	6.4	7.0	8.0	3.0	4.9	5.0	7.0	3.0	5.3	6.0	7.0
Sparkling rosé	5.0	5.9	6.0	7.0	3.0	4.4	5.0	5.5	5.0	6.1	7.0	7.0	5.0	6.1	6.0	7.0
Sparkling red	4.0	5.8	6.0	8.0	3.0	5.3	5.0	7.0	3.0	5.6	6.0	8.0	5.0	6.4	7.0	8.0
Moscato	3.0	4.8	5.0	7.0	2.0	4.1	3.0	6.0	3.0	5.3	6.0	7.0	2.0	4.4	3.5	7.0
Prosecco	4.0	5.7	6.0	7.0	2.8	5.2	5.0	7.3	4.0	5.6	6.0	7.0	5.0	5.9	6.0	7.3
**Mood Medium Test Multiple Pairwise Comparison *p* Values**																
	**All Consumers**				**No Frills vs Aspirants**				**No Frills vs Enthusiasts**				**Aspirants vs Enthusiasts**			
Champagne	0.256				0.125				0.762				0.326			
Sparkling white (CA)	0.108				0.719				0.117				0.065			
Sparkling white (CH)	0.294				0.004 *				0.185				0.190			
Sparkling white (TR)	0.637				0.936				0.490				0.374			
Sparkling white (MT)	0.073				0.025 *				0.233				0.239			
Sparkling rosé	0.032 *				0.009 *				0.058				0.548			
Sparkling red	0.201				0.622				0.429				0.538			
Moscato	0.037 *				0.080				0.866				0.022 *			
Prosecco	0.978				0.979				0.867				0.853			

Data represent mean, median and quartiles of 9-point hedonic scale scores (where 1 = extremely dislike, 5 – neither dislike or like, and 9 = extremely like). * denotes P values at ≤ 0.05. CA = carbonated; CH = Charmat; TR = Transfer; MT = Méthode Traditionelle.

**Table 6:** Consumers’ and Fine Wine Instrument consumer segments’ knowledge of different sparkling wine production methods.

**1st Percentage Quartile | Mean | Median | 3rd Percentage Quartile**																
	**All Consumers** **(*n* = 203)**				**No Frills** **(*n* = 31)**				**Aspirants** **(*n* = 104)**				**Enthusiast** **(*n* = 68)**			
Champagne	1.0	3.6	3.0	6.5	1.0	1.4	1.0	1.0	1.0	3.5	2.0	6.0	2.0	4.6	5.0	7.0
Sparkling white (CA)	1.0	3.2	2.0	5.0	1.0	1.5	1.0	2.0	1.0	3.1	2.0	5.0	1.0	4.1	3.0	7.0
Sparkling white (CH)	1.0	1.9	1.0	2.0	1.0	1.0	1.0	1.0	1.0	1.8	1.0	2.0	1.0	2.4	1.0	3.0
Sparkling white (TR)	1.0	2.0	1.0	2.0	1.0	1.1	1.0	1.0	1.0	2.0	1.0	2.0	1.0	2.3	1.0	3.0
Sparkling white (MT)	1.0	3.1	2.0	5.0	1.0	1.3	1.0	1.0	1.0	2.9	2.0	5.0	2.0	4.2	3.5	7.0
**Mood Medium Test Multiple Pairwise Comparison *p* Values**																
	**All Consumers**				**No Frills vs Aspirants**				**No Frills vs Enthusiasts**				**Aspirants vs Enthusiasts**			
Champagne	<0.0001 *				0.001 *				<0.0001 *				0.025 *			
Sparkling white (CA)	<0.0001 *				0.020 *				<0.0001 *				0.058			
Sparkling white (CH)	0.001 *				0.001 *				<0.0001 *				0.341			
Sparkling white (TR)	0.001 *				0.001 *				<0.001 *				0.629			
Sparkling white (MT)	<0.0001 *				<0.0001 *				<0.0001 *				0.001 *			

Data represent mean, median and quartiles of 9-point Likert scale scores (where 1 = extremely unfamiliar, 5 = neither unfamiliar nor familiar, and 9 = extremely familiar). * denotes *p* values at ≤0.05. CA = carbonated; CH = Charmat; TR = Transfer; MT = Méthode Traditionelle.

**Table 7:** Ability of consumers and Fine Wine Instrument consumer segments to correctly identify different sparkling wine styles (during blind tastings).

	Proportion ^a^ | Percentage							
	All Consumers		No Frills		Aspirants		Enthusiast	
Champagne	26/137	19.0	4/17	23.5	14/77	18.2	8/43	18.6
Sparkling white (CA)	12/135	8.9	3/21	14.3	4/68	5.9	5/46	10.9
Sparkling white (CH)	5/136	3.7	1/21	4.8	2/65	3.1	2/50	4.0
Sparkling white (TR)	3/134	2.2	0/19	0.0	2/74	2.7	1/41	2.4
Sparkling white (MT)	12/132	9.1	2/21	9.5	5/67	7.5	5/44	11.4
Sparkling rosé	5/139	3.6	0/19	0.0	2/77	2.6	3/43	7.0
Sparkling red	77/130	59.2	12/21	57.1	34/61	55.7	31/48	64.6
Moscato	109/137	79.6	19/23	82.6	58/70	82.9	32/44	72.7
Prosecco	8/138	5.8	1/25	4.0	2/65	3.1	5/48	10.4

Champagne was the only sparkling wine style for which responses were statistically significant (at *p* ≤ 0.003 for all segments, *k* Proportions Test, and at *p* ≤ 0.05 for No Frills vs. Enthusiasts, Marascuilo Procedure). CA = carbonated; CH = Charmat; TR = Transfer; MT = Méthode Traditionelle. ^a^ Consumers who correctly identified a sparkling wine, relative to consumers who tasted that sparkling wine.

**Table 8:** Comparison of expected and actual liking scores of consumers and Fine Wine Instrument consumer segments for different sparkling wine styles.

	**Survey (Expected) Median Liking | Tasting (Actual) Median Liking**							
	**All Consumers**		**No Frills**		**Aspirants**		**E** **nthusiast**	
Champagne	9.0	6.0	7.5	7.0	8.0	6.0	9.0	7.0
Sparkling white (MT)	8.0	6.0	9.0	7.0	8.0	5.0	8.0	6.0
Sparkling rosé	6.0	6.0	6.0	5.0	7.0	7.0	5.0	6.0
Sparkling red	7.0	6.0	7.0	5.0	7.0	6.0	8.0	7.0
Moscato	5.0	5.0	5.5	3.0	5.5	6.0	4.0	3.5
Prosecco	5.0	6.0	5.0	5.0	5.5	6.0	5.5	6.0
	**Wilcoxon Signed-Rank Test *p*-Value**							
Champagne	<0.0001 *		0.201		<0.0001 *		<0.0001 *	
Sparkling white (MT)	<0.0001 *		0.004 *		<0.0001 *		<0.0001 *	
Sparkling rosé	0.919		0.087		0.932		0.126	
Sparkling red	<0.0001 *		0.252		0.003 *		0.015 *	
Moscato	0.189		0.028 *		0.636		0.895	
Prosecco	0.447		0.479		0.625		0.850	

Data represent medians of 9-point hedonic scale scores (where 1 = extremely dislike, 5 = neither dislike or like, and 9 = extremely like). * denotes *p* values at ≤0.05.

**Table 9:** Typical spend by consumers and Fine Wine Instrument consumer segments for different sparkling wine styles (as Australian dollars per 750 mL bottle).

		Survey Frequency | Tasting Frequency												Fisher Exact *p* Value		
		Never Purchase		<$15		$15–29		$30–49		$50–79		>$80		Survey|Tasting Segment × Price		Survey & Tasting × Price
Champagne (*n* = 137)	All Consumers	17	26	7	31	25	48	41	22	41	7	6	3	0.005 *	0.617	1.000
	No Frills	5	3	4	6	4	5	1	3	2	0	1	0			0.799
	Aspirants	9	17	1	16	15	23	24	15	24	4	4	2			1.000
	Enthusiasts	3	6	2	9	6	20	16	4	15	3	1	1			1.000
Sparkling white (MT) (*n* = 132)	All Consumers	2	44	29	33	80	34	18	17	3	3	0	1	1.000	1.000	1.000
	No Frills	1	3	7	7	12	8	1	3	0	0	0	0			1.000
	Aspirants	1	26	16	13	41	18	8	9	1	1	0	0			1.000
	Enthusiasts	0	15	6	13	27	8	9	5	2	2	0	1			1.000
Sparkling rosé (*n* = 139)	All Consumers	51	28	25	38	60	57	3	13	0	3	0	0	0.343	0.048 *	1.000
	No Frills	10	10	4	5	5	4	0	0	0	0	0	0			0.880
	Aspirants	27	14	16	19	33	34	1	8	0	2	0	0			0.980
	Enthusiasts	14	4	5	14	22	19	2	5	0	1	0	0			1.000
Sparkling red (*n* = 130)	All Consumers	31	35	22	27	50	48	21	18	6	2	0	0	0.059	0.106	1.000
	No Frills	10	11	4	4	6	4	1	2	0	0	0	0			0.959
	Aspirants	15	16	11	14	24	24	10	7	1	0	0	0			0.120
	Enthusiasts	6	8	7	9	20	20	10	9	5	2	0	0			0.390
Moscato (*n* = 137)	All Consumers	66	63	36	38	30	33	5	3	0	0	0	0	0.307	0.295	0.156
	No Frills	15	14	4	5	3	4	1	0	0	0	0	0			0.440
	Aspirants	28	27	20	24	18	16	4	3	0	0	0	0			0.014 *
	Enthusiasts	23	22	12	9	9	13	0	0	0	0	0	0			0.017 *
Prosecco (*n* = 138)	All Consumers	93	35	17	33	25	53	3	14	0	3	0	0	<0.0001 *	0.140	1.000
	No Frills	24	11	1	5	0	9	0	0	0	0	0	0			1.000
	Aspirants	43	15	12	13	7	29	3	7	0	1	0	0			1.000
	Enthusiasts	26	9	4	15	18	15	0	7	0	2	0	0			1.000

* denotes *p* values at ≤ 0.05.

**Table 10:** Comparison of Fine Wine Instrument consumer segment preferences for different sparkling wine styles by occasion.

		**Wilcoxon-Signed Rank Test *p* Values**								
		**Anniversary**	**At Home** **with Food**	**At Home** **without Food**	**Birthday**	**Breakfast**	**By Yourself**	**Christmas**	**Funeral**	**Girls’ or** **Boys’ Night**
Champagne	No Frills	0.442	0.386	0.285	0.899	0.724	0.730	0.688	0.041 *	0.720
	Aspirants	0.005 *	0.148	0.908	0.109	0.990	0.140	0.022 *	0.648	0.765
	Enthusiasts	0.005 *	0.947	0.540	0.137	0.734	0.545	0.023 *	0.104	0.820
Sparkling white	No Frills	0.053	0.836	0.268	0.030 *	0.072	0.435	0.004 *	0.832	0.412
	Aspirants	<0.0001 *	0.020 *	0.028 *	<0.0001 *	0.571	0.835	<0.0001 *	0.039 *	0.001 *
	Enthusiasts	<0.0001 *	0.001 *	0.004 *	<0.0001 *	0.003 *	0.939	<0.0001 *	0.534	0.104
Sparkling rosé	No Frills	0.482	0.083	0.272	0.779	0.050 *	0.410	0.636	0.105	0.858
	Aspirants	<0.0001 *	0.010 *	0.000 *	<0.0001 *	<0.0001 *	0.000 *	<0.0001 *	0.000 *	0.000 *
	Enthusiasts	<0.0001 *	0.014 *	0.001 *	<0.0001 *	0.001 *	0.008 *	<0.0001 *	0.006 *	0.000 *
Sparkling red	No Frills	0.384	0.725	0.622	0.906	1.000	0.440	0.751	0.174	0.677
	Aspirants	0.814	0.325	0.264	0.358	0.074	0.107	0.426	0.014 *	0.386
	Enthusiasts	0.066	0.006 *	0.190	0.001 *	0.149	0.030 *	0.360	0.001 *	0.260
Moscato	No Frills	0.393	0.019 *	0.136	0.548	0.766	1.000	0.003 *	1.000	0.066
	Aspirants	0.004 *	0.022 *	0.013 *	0.006 *	0.000 *	0.076	0.000 *	0.018 *	0.025 *
	Enthusiasts	0.008 *	0.004 *	0.039 *	0.090	0.041 *	0.008 *	0.036 *	0.013 *	0.032 *
Prosecco	No Frills	0.000 *	0.002 *	0.002 *	0.000 *	0.002 *	0.005 *	0.000 *	0.005 *	0.001 *
	Aspirants	<0.0001 *	0.001 *	0.001 *	<0.0001 *	0.000 *	0.001 *	<0.0001 *	0.000 *	<0.0001 *
	Enthusiasts	<0.0001 *	0.000 *	0.000 *	<0.0001 *	0.001 *	0.001 *	<0.0001 *	0.001 *	<0.0001 *
		**Hot Weather**	**Melbourne** **Cup**	**New Year**	**Pub or Club**	**Restaurant** **or Café**	**Weddings**	**Weekdays**	**Weekends**	**Work Drinks**
Champagne	No Frills	0.621	0.301	0.263	0.148	0.566	0.146	0.648	0.089	0.693
	Aspirants	0.346	0.573	0.010 *	0.220	0.013 *	0.000 *	0.821	0.965	0.010 *
	Enthusiasts	0.472	0.311	0.002 *	0.922	0.172	0.007 *	0.991	0.993	0.114
Sparkling white	No Frills	0.178	0.347	0.032 *	0.190	1.000	0.019 *	0.949	0.343	0.969
	Aspirants	0.000	0.001 *	<0.0001 *	0.010 *	0.003 *	<0.0001 *	0.053	0.001 *	0.097
	Enthusiasts	0.001 *	0.001 *	<0.0001 *	0.043 *	0.001 *	<0.0001 *	0.004 *	<0.0001 *	0.034 *
Sparkling rosé	No Frills	0.888	0.437	0.259	0.796	0.499	0.169	0.102	0.220	0.632
	Aspirants	0.050 *	<0.0001 *	<0.0001 *	<0.0001 *	<0.0001 *	<0.0001 *	0.036 *	0.001 *	<0.0001 *
	Enthusiasts	0.000 *	<0.0001 *	<0.0001 *	0.003 *	0.000 *	<0.0001 *	0.009 *	<0.0001 *	<0.0001 *
Sparkling red	No Frills	0.918	0.106	0.526	0.858	0.607	0.861	1.000	0.753	0.108
	Aspirants	0.495	0.580	0.409	0.888	0.453	0.187	0.430	0.716	0.006 *
	Enthusiasts	0.047 *	0.003 *	0.429	0.012 *	0.003 *	<0.0001 *	0.037 *	0.020 *	0.025 *
Moscato	No Frills	0.181	0.022 *	0.010 *	0.203	0.106	0.046 *	0.219	0.548	0.041 *
	Aspirants	0.080 *	0.001 *	0.000 *	0.018 *	0.001 *	<0.0001 *	0.066	0.012 *	0.000 *
	Enthusiasts	0.039 *	0.000 *	0.001 *	0.043 *	0.027 *	0.003 *	0.007 *	0.010 *	0.119
Prosecco	No Frills	0.002 *	0.000 *	0.001 *	0.001 *	<0.0001 *	0.000 *	0.001 *	0.000 *	0.001 *
	Aspirants	0.000 *	<0.0001 *	<0.0001 *	<0.0001 *	0.002 *	<0.0001 *	0.000 *	<0.0001 *	<0.0001 *
	Enthusiasts	<0.0001 *	<0.0001 *	<0.0001 *	<0.0001 *	<0.0001 *	<0.0001 *	0.002 *	0.000 *	0.000 *

* denotes *p* values at ≤ 0.05.

## Supplementary Materials

The following are available online at https://www.mdpi.com/2304-8158/10/3/488/s1, Table S1: Attributes and standards used in descriptive analysis of sparkling wines, Table S2: Mean intensity ratings for sensory attributes of the French Champagne and Australian sparkling wines studied, Table S3: Alcohol, wine and sparkling wine consumption of consumers and of Fine Wine Instrument consumer segments. Data represent minimum, mean, median and maximum responses (on a percentage scale, i.e., 0–100%), Table S4: Influence of occasion on consumers’ and Fine Wine Instrument consumer segments’ consumption of different sparkling wine styles, Table S5: Statistical analysis for influence of occasion on consumers’ and Fine Wine Instrument consumer segments’ consumption of different sparkling wine styles.

## References

[1] Wine Australia (2019) Will Sparkling Wine’s Bubble Continue to Rise?. https://www.wineaustralia.com/news/market-bulletin/issue-186.

[2] Verdonk N., Culbert J., Wilkinson K. (2015). Sparkling wine: All that sparkles: Consumer perceptions of sparkling wine. Wine Vitic. J..

[3] Culbert J., Ristic R., Ovington L., Saliba A., Wilkinson K. (2017). Influence of production method on the sensory profile and consumer acceptance of Australian sparkling white wine styles. Aust. J. Grape Wine Res..

[4] Culbert J., Ristic R., Ovington L., Saliba A., Wilkinson K. (2018). Sensory profiles and consumer acceptance of different styles of Australian Moscato. Aust. J. Grape Wine Res..

[5] Iland P., Gago P. (1997). Australian Wine: From the Vine to the Glass.

[6] Alexandre H., Guilloux-Benatier M. (2006). Yeast autolysis in sparkling wine—A review. Aust. J. Grape Wine Res..

[7] Culbert J., Verdonk N., Ristic R., Olarte Mantilla S., Lane M., Pearce K., Cozzolino D., Wilkinson K. (2016). Understanding consumer preferences for Australian sparkling wine vs. French Champagne. Beverages.

[8] Gluckman R. (1990). A consumer approach to branded wines. Int. J. Wine Mark..

[9] Keown C., Casey M. (1995). Purchasing behaviour in the northern Ireland wine market. Br. Food J..

[10] Chaney I. (2000). External search effort for wine. Int. J. Wine Mark..

[11] Charters S. (1993). Drinking sparkling wine: An exploratory investigation. Int. J. Wine Mark..

[12] Charters S., Pettigrew S. (2007). The dimensions of wine quality. Food Qual. Prefer..

[13] Lesschaeve I., Bowen A., Bruwer J. (2012). Determining the impact of consumer characteristics to project sensory preferences in commercial white wines. Am. J. Enol. Vitic..

[14] Melo L., Delahunty C., Cox D.N. (2011). A new approach using consumers ‘drinking histories’ to explain current wine acceptance. Food Res. Int..

[15] Vecchio R., Lisanti M., Caracciolo F., Cembalo L., Gambuti A., Moio L., Siani T., Marotta G., Nazzaro C., Piombino P. (2019). The role of production process and information on quality expectations and perceptions of sparkling wines. J. Sci. Food Agric..

[16] Harrar V., Smith B., Deroy O., Spence C. (2013). Grape expectations: How the proportion of white grape in Champagne affects the ratings of experts and social drinkers in a blind tasting. Flavour.

[17] McMahon K.M., Culver C., Ross C.F. (2017). The production and consumer perception of sparkling wines of different carbonation levels. J. Wine Res..

[18] McMahon K.M., Diako C., Aplin J., Mattinson D.S., Culver C., Ross C.F. (2017). Trained and consumer panel evaluation of sparkling wines sweetened to brut or demi sec residual sugar levels with three different sugars. Food Res. Int..

[19] Hood White M.R., Heymann H. (2015). Assessing the sensory profiles of sparkling wine over time. Am. J. Enol. Vitic..

[20] Polidori G., Jeandet P., Liger-Belair G. (2009). Bubbles and flow patterns in Champagne: Is the fizz just for show, or does it add to the taste of sparkling wines?. Am. Sci..

[21] Lange C., Martin C., Chabanet C., Combris P., Issanchou S. (2002). Impact of the information provided to consumers on their willingness to pay for Champagne: Comparison with hedonic scores. Food Qual. Prefer..

[22] Combris P., Lange C., Issanchou S. (2006). Assessing the effect of information on the reservation price for Champagne: What are consumers actually paying for?. J. Wine Econ..

[23] Verdonk N., Wilkinson J., Culbert J., Ristic R., Pearce K., Wilkinson K. (2017). Toward a model of sparkling wine purchasing preferences. Int. J. Wine Bus. Res..

[24] Velikova N., Charters S., Fountain J., Ritchie C., Fish N., Dodd T. (2016). Status or fun? A cross-cultural examination of young consumers’ responses to images of champagne and sparkling wine. Brit. Food J..

[25] Cerjak M., Tomić M., Fočić N., Brkić R. (2016). The importance of intrinsic and extrinsic sparkling wine characteristics and behavior of sparkling wine consumers in Croatia. J. Int. Food Agribus. Mark..

[26] Morton A., Healy M., Rivers C. Beyond the bubbles: Identifying other purchase decision variables beyond country of origin effect that make Australians buy Champagne. Proceedings of the Australia-New Zealand International Business Association Conference: Dynamism and Challenges in Internationalisation.

[27] Verdonk N., Ristic R., Culbert J., Pearce K., Wilkinson K. (2020). Understanding Australian wine consumers’ preferences for different sparkling wine styles. Beverages.

[28] Morton A., Rivers C., Charters S., Spinks W. (2013). Champagne purchasing: The influence of kudos and sentimentality. Qual. Mark. Res..

[29] Rao A., Monroe K. (1989). The effect of price, brand name, and store name on buyers’ perceptions of product quality: An integrative review. J. Mark. Res..

[30] Spawton A. (1993). Grapes and wine seminar—prospering in the 1990s: Changing your view of the consumer. Int. J. Wine Mark..

[31] Skuras D., Vakrou A. (2002). Consumers’ willingness to pay for origin labelled wine: A Greek case study. Br. Food J..

[32] Schamel G., Anderson K. (2003). Wine quality and varietal, regional and winery reputations: Hedonic prices for Australia and New Zealand. Econ. Rec..

[33] Jover A., Montes F., del Mar Fuentes M. (2004). Measuring perceptions of quality in food products: The case of red wine. Food Qual. Prefer..

[34] Charters S., Velikova N., Ritchie C., Fountain J., Thach L., Dodd T., Fish N., Herbst F., Terblanche N. (2011). Generation Y and sparkling wines: A cross-cultural perspective. Int. J. Wine Bus. Res..

[35] Fountain J., Lamb C. (2011). Generation Y as young wine consumers in New Zealand: How do they differ from generation x?. Int. J. Wine Bus. Res..

[36] Pickering G.J., Cullen C.W. The influence of taste sensitivity and adventurousness on Generation Y’s liking scores for sparkling wine. Proceedings of the 4th AWBR International Conference.

[37] Polymeros C., Athanasios K., Ana M., Rachel L. (2012). Generation Y preferences for wine: An exploratory study of the US market applying the best-worst scaling. Br. Food J..

[38] Qenani-Petrela E., Wolfe M., Zuckerman B. (2007). Generational differences in wine consumption. J. Food Distrib. Res..

[39] Thach L. Wine for breakfast: Exploring wine occasions for Gen Y. Proceedings of the 6th AWBR International Conference.

[40] Lerro M., Vecchio R., Nazzaro C., Pomarici E. (2019). The growing (good) bubbles: Insights into US consumers of sparkling wine. Br. Food J..

[41] Bruwer J., Saliba A., Miller B. (2011). Consumer behaviour and sensory preference differences: Implications for wine product marketing. J. Consum. Mark..

[42] Barber N., Almanza B.A., Donovan J.R. (2006). Motivational factors of gender, income and age on selecting a bottle of wine. Int. J. Wine Mark..

[43] Sharon L.F. (2012). The influence of gender on wine purchasing and consumption: An exploratory study across four nations. Int. J. Wine Bus. Res..

[44] Thach L. (2012). Time for wine? Identifying differences in wine-drinking occasions for male and female wine consumers. J. Wine Res..

[45] Johnson T., Bastian S. (2015). A fine wine instrument–an alternative for segmenting the Australian wine market. Int. J. Wine Bus. Res..

[46] Beverland M. (2006). The ‘real thing’: Branding authenticity in the luxury wine trade. J. Bus. Res..

[47] Culbert J., Cozzolino D., Ristic R., Wilkinson K. (2015). Classification of sparkling wine style and quality by MIR spectroscopy. Molecules.

[48] Iland P. (2004). Chemical Analysis of Grapes and Wine: Techniques and Concepts.

[49] Lawless H.T., Heymann H. (2010). Descriptive analysis. In Sensory Evaluation of Food: Principles and Practices.

[50] Gawel R., Godden P.W. (2008). Evaluation of the consistency of wine quality assessments from expert wine tasters. Aust. J. Grape Wine Res..

[51] Thiene M., Scarpa R., Galletto L., Boatto V. (2013). Sparkling wine choice from supermarket shelves: The impact of certification of origin and production practices. Agric. Econ..

[52] Rossetto L., Gastaldello G. (2018). The loyalty structure of sparkling wine brands in Italy. J. Wine. Econ..

[53] Kallas Z., Escobar C., Gil J.M. (2012). Assessing the impact of a Christmas advertisement campaign on Catalan wine preference using choice experiments. Appetite.

[54] Mueller Loose S., Jaeger S.R. (2012). Factors that influence beverage choices at meal times. An application of the food choice kaleidoscope framework. Appetite.

[55] Viot C. (2012). Subjective knowledge, product attributes and consideration set: A wine application. Int. J. Wine Bus. Res..

