# Supplementary material for: Investigating Australian Consumers’ Perceptions of and Preferences for Different Styles of Sparkling Wine Using the Fine Wine Instrument
Source: Foods. 2021 Feb 24;10(3):488. doi: 10.3390/foods10030488 (PMC7996294; doi:10.3390/foods10030488)
Supplement: Supplementary file 1 [file foods-10-00488-s001.pdf]

foods

MDPI

Supplementary Materials

Article

Investigating Australian Consumers' Perceptions of and Preferences for Different Styles of Sparkling Wine Using the Fine Wine Instrument

Naomi Verdonk[1], Renata Ristic[1], Julie Culbert[1,†], Karma Pearce[2] and Kerry Wilkinson[1,*]

[1] School of Agriculture, Food and Wine, The University of Adelaide, Waite Campus, PMB 1, Glen Osmond, SA, 5064, Australia; naomi.verdonk@adelaide.edu.au (N.V.); renata.ristic@adelaide.edu.au (R.R.); julie.culbert@adelaide.edu.au (J.C.)
[2] School of Pharmacy and Medical Sciences, University of South Australia, GPO Box 2471, Adelaide, SA, 5001, Australia; karma.pearce@unisa.edu.au (K.P.)
† Current address: The Australian Wine Research Institute, PO Box 197, Glen Osmond, SA, 5064, Australia.
* Correspondence: kerry.wilkinson@adelaide.edu.au (K.W.); Tel.: +61-8-8313-7360

Table S1. Attributes and standards used in descriptive analysis of sparkling wines.

Attributes	Reference standards
apple/pear	⅛ green pear, ⅛ green apple
bruised apple	50 mL dry sherry, ⅛ green apple
citrus	⅛ yellow grapefruit, ⅛ orange, ⅛ lemon, ⅛ lime
confectionary	3 red frog sweets, 1 strawberry and cream sweet
dark fruit	5 tsp Ribena cordial, 5 tsp four berry jam, 1 tsp mulberry extract
floral/musk	2 red rose petals, ½ tsp rose water, 1 musk stick
honey	2 tsp honey
mixed spice	½ cinnamon stick, ½ tsp mixed spice
oaky	10 oak chips
toasty/nutty	2 mini toasts, 3 water crackers, 1 tbs mixed nuts
savory/smoky	½ bacon rasher, 2 tsp smoked almonds
stone fruit	2 dried apricots, 1 dried peach, ½ canned peach, 1 canned apricot
tropical fruit	⅛ rock melon, ½ lychee, ⅛ passionfruit, ⅛ pineapple, ⅛ mango
vanilla/caramel	2 tsp desiccated coconut, ½ tsp vanilla essence
yeasty	1 tbs wine yeast

Standards were prepared in 25 mL of Chardonnay wine, except dark fruit which was prepared in 25 mL of Shiraz wine.

Table S2. Mean intensity ratings for sensory attributes of the French Champagne and Australian sparkling wines studied.

Attribute	Champagne	Sparkling white (CA)	Sparkling white (CH)	Sparkling white (TR)	Sparkling white (MT)	Sparkling red	Sparkling rosé	Moscato	Prosecco
overall intensity A	9.38 bcd	7.84 d	8.86 bcd	9.51 bc	9.54 bc	10.32 ab	8.44 cd	11.95 a	9.45 bcd
apple/pear A	5.1 a	5.6 a	6.3 a	5.3 a	5.6 a	2.4 b	5.3 a	5.0 a	5.7 a
bruised apple A	4.8 b	3.0 bc	1.9 c	7.9 a	7.6 a	1.9 c	4.1 b	1.3 c	1.5 c
citrus A	5.9 a	5.7 a	7.0 a	5.0 a	5.7 a	1.7 b	5.1 a	6.2 a	6.5 a
confectionary A	1.9 e	3.2 cde	5.3 bc	2.7 de	3.2 cde	4.4 bcd	3.5 cde	9.5 a	6.7 b
dark fruit A	0.6 b	0.9 b	1.1 b	0.7 b	0.6 b	10.9 a	0.7 b	1.4 b	1.3 b
floral/musk A	2.2 c	2.7 c	6.2 b	3.5 c	2.9 c	3.5 c	2.7 c	12.2 a	7.5 b
honey A	3.3 bc	3.2 bc	4.1 abc	4.7 ab	4.5 abc	2.5 c	4.0 abc	5.4 a	4.4 abc
mixed spice A	1.6 b	1.2 b	1.0 b	1.1 b	1.4 b	6.7 a	1.3 b	1.9 b	1.2 b
oaky A	2.0 b	1.6 bc	1.0 bc	1.3 bc	1.3 bc	9.3 a	1.5 bc	0.6 c	0.7 bc
savoury/smoky A	4.0 ab	2.7 bc	1.4 cd	2.2 cd	2.1 cd	4.8 a	2.8 bc	0.9 d	1.3 cd
stone fruit A	4.1 cd	4.2 bcd	6.5 ab	4.5 bc	4.9 bc	2.0 d	4.9 bc	8.4 a	7.2 a
toasty/nutty A	6.2 a	3.0 cde	2.2 de	5.3 ab	5.5 ab	4.3 abc	3.8 bcd	1.2 e	1.5 e
tropical fruit A	3.1 de	4.4 cd	6.1 bc	4.3 cd	3.6 de	1.5 e	3.8 de	9.1 a	7.6 ab
vanilla/caramel A	2.3 b	2.5 b	2.4 b	2.4 b	3.0 b	5.0 a	2.9 b	2.7 b	2.7 b
yeasty A	5.6 a	2.6 cd	1.9 cd	5.1 ab	5.0 ab	2.5 cd	3.3 bc	0.9 d	1.4 d
overall intensity F	9.60 bcd	9.18 cd	8.76 d	9.44 cd	10.33 bc	10.76 ab	9.72 bcd	11.73 a	9.32 cd
apple/pear F	6.2 a	6.4 a	6.8 a	6.1 a	6.6 a	2.1 b	6.0 a	5.7 a	6.1 a
bruised apple F	5.5 ab	3.5 bc	3.0 c	7.2 a	7.4 a	1.7 c	5.4 ab	1.2 c	2.1 c
citrus F	8.4 a	8.4 a	8.6 a	8.4 a	7.8 a	1.9 b	8.2 a	6.6 a	7.8 a
confectionary F	2.0 d	3.3 cd	4.9 bc	2.0 d	2.5 d	4.7 bc	2.9 cd	10.1 a	5.7 b
dark fruit F	0.7 b	0.7 b	0.9 b	0.7 b	0.4 b	11.4 a	0.6 b	1.4 b	0.8 b
floral/musk F	2.4 d	3.0 d	4.8 c	2.7 d	2.3 d	2.9 d	2.7 d	12.0 a	7.0 b

honey F	3.1 bc	2.8 bc	4.0 ab	3.7 abc	3.9 abc	2.0 c	3.3 bc	5.5 a	3.7 abc
mixed spice F	1.4 b	1.1 b	1.2 b	0.9 b	0.9 b	7.4 a	1.2 b	2.1 b	1.4 b
oaky F	2.0 b	1.6 bc	0.9 bc	1.0 bc	1.5 bc	10.0 a	1.8 bc	0.6 c	1.0 bc
savoury/smoky F	2.8 ab	2.0 bc	1.4 bc	1.6 bc	2.0 bc	3.6 a	2.3 abc	0.9 c	1.2 c
stone fruit F	4.3 c	4.9 bc	6.5 abc	4.3 c	4.6 bc	1.8 d	4.6 bc	8.7 a	6.6 ab
toasty/nutty F	5.5 a	2.5 bc	2.2 bc	4.6 a	4.7 a	3.9 ab	4.8 a	1.1 c	1.5 c
tropical F	3.9 de	4.7 cd	6.2 bc	4.2 cd	3.7 de	1.6 e	4.4 cd	9.4 a	7.6 ab
vanilla/caramel F	2.0 b	1.6 b	2.2 b	2.1 b	2.6 b	4.6 a	2.4 b	2.4 b	2.5 b
yeasty F	5.1 a	2.3 cd	2.7 bcd	4.4 ab	5.1 a	2.3 cd	4.1 abc	1.0 d	2.3 cd
effervescence	3.38 b	4.63 ab	4.84 ab	4.32 ab	3.53 b	6.42 a	4.09 ab	4.85 ab	5.52 ab
acidity	10.21 a	10.56 a	9.90 a	10.19 a	10.08 a	6.79 b	10.81 a	3.04 c	7.23 b
bitterness	4.87 b	3.79 b	3.32 bc	4.39 b	4.44 b	9.10 a	4.71 b	1.11 c	4.27 b
sweetness	1.84 c	2.36 c	2.83 c	1.90 c	2.05 c	4.53 b	2.06 c	13.19 a	4.55 b
astringency	5.09 b	4.55 b	3.48 bc	4.91 b	4.63 b	10.69 a	4.75 b	1.65 c	3.64 b
complexity	8.54 a	5.91 bcd	5.46 cd	8.29 a	8.52 a	7.57 ab	6.93 abc	2.37 e	4.29 de

A = aroma attributes; F = flavor attribute; MT = Méthode Traditionelle; TR = Transfer; CH = Charmat; CA = carbonated. Values are means from three wine replicates, presented to 12 judges during three formal sensory evaluation sessions. Means followed by different letters (within rows) are significantly different at $P < 0.001$ ($P \leq 0.05$, one-way ANOVA, Tukey's LSD post hoc).

Table S3. Alcohol, wine and sparkling wine consumption of consumers and of Fine Wine Instrument consumer segments. Data represent minimum, mean, median and maximum responses (on a percentage scale, i.e., 0–100%).

	**All Consumers (*n* = 203)**				**No Frills (*n* = 31)**				**Aspirants (*n* = 104)**				**Enthusiast (*n* = 68)**			
Wine	10.0	68.2	75.0	100.0	10.0	67.8	80.0	90.0	15.0	66.9	75.0	100.0	20.0	70.5	75.0	100.0
Beer	0.0	18.9	10.0	80.0	0.0	14.8	10.0	80.0	0.0	19.9	10.0	75.0	0.0	18.9	15.0	60.0
Spirits	0.0	12.3	10.0	70.0	0.0	17.6	10.0	70.0	0.0	12.4	10.0	60.0	0.0	9.9	10.0	40.0
Cider	0.0	7.4	5.0	90.0	0.0	13.3	5.0	90.0	0.0	6.6	4.5	40.0	0.0	6.7	5.0	30.0
Other	0.0	3.3	0.0	40.0	0.0	4.2	0.0	25.0	0.0	3.3	0.0	40.0	0.0	3.0	0.0	15.0
Sparkling wine	2.0	23.2	20.0	94.0	5.0	37.1	25.0	89.0	2.0	21.7	20.0	75.0	5.0	19.1	18.5	55.0
White	0.0	28.6	25.0	90.0	0.0	32.9	25.0	85.0	0.0	27.4	25.0	90.0	0.0	28.5	25.0	80.0
Rose	0.0	9.4	5.0	50.0	0.0	11.3	10.0	25.0	0.0	9.8	5.0	50.0	0.0	8.1	5.0	40.0
Red	0.0	42.4	40.0	90.0	0.0	32.5	22.5	80.0	0.0	43.0	40.0	90.0	5.0	44.9	42.5	90.0
Dessert	0.0	3.8	2.0	30.0	0.0	5.4	3.5	25.0	0.0	3.1	1.0	20.0	0.0	4.6	5.0	30.0
Fortified	0.0	4.1	4.5	20.0	0.0	3.8	5.0	10.0	0.0	3.7	2.0	20.0	0.0	4.8	5.0	15.0
Champagne	0.0	14.5	10.0	100.0	0.0	10.5	5.0	100.0	0.0	12.0	10.0	60.0	0.0	14.9	10.0	70.0
Sparkling white	0.0	53.8	55.0	100.0	0.0	58.5	72.5	100.0	0.0	50.7	50.0	100.0	0.0	52.6	50.0	100.0
Sparkling red	0.0	22.4	15.0	100.0	0.0	13.5	9.0	90.0	0.0	20.3	20.0	100.0	0.0	16.9	10.0	70.0
Sparkling rose	0.0	8.9	5.0	65.0	0.0	5.8	5.0	65.0	0.0	5.8	5.0	40.0	0.0	5.1	5.0	50.0
Moscato	0.0	11.5	5.0	100.0	0.0	9.8	5.0	100.0	0.0	7.0	5.0	60.0	0.0	4.4	5.0	60.0
Prosecco	0.0	8.0	3.0	65.0	0.0	1.9	0.0	30.0	0.0	4.2	0.0	60.0	0.0	6.1	5.0	65.0

Mood's Medium Test Multiple Pairwise Comparison P values				
	**All Consumers**	**No Frills vs Aspirants**	**No Frills vs Enthusiasts**	**Aspirants vs Enthusiasts**
Wine	0.866	0.593	0.674	0.907
Beer	0.346	0.228	0.148	0.621
Spirits	0.847	0.650	0.566	0.838
Cider	0.965	0.864	0.991	0.809
Other	0.761	0.897	0.708	0.461
Sparkling wine	<0.001*	0.006*	<0.001*	0.063
White	0.938	0.997	0.807	0.733
Rose	0.281	0.269	0.112	0.425
Red	0.760	0.576	0.460	0.751
Dessert	0.177	0.145	0.760	0.065
Fortified	0.131	0.340	0.825	0.030*
Champagne	0.526	0.636	0.595	0.471
Sparkling white	0.237	0.080	0.039*	0.921
Sparkling red	0.026*	0.186	0.286	0.012*
Sparkling rose	0.880	0.849	0.875	0.616
Moscato	0.290	0.811	0.216	0.147
Prosecco	0.052	0.868	0.373	0.017*

* denotes P values at ≤ 0.05.

Table S4. Influence of occasion on consumers' and Fine Wine Instrument consumer segments' consumption of different sparkling wine styles.

		Survey Median I Tasting Median																	
		**Anniversary**		**At home with food**		**At home without food**		**Birthday**		**Breakfast**		**By yourself**		**Christmas**		**Funeral**		**Girls' or boys' night**	
Champagne (*n* = 137)	All Consumers	5.0	4.0	3.0	3.0	3.0	2.0	5.0	5.0	2.0	2.0	1.0	1.0	6.0	5.0	1.0	1.0	2.0	2.0
	No Frills	3.0	5.0	2.0	3.0	2.0	3.0	5.0	5.0	2.0	1.0	1.0	1.0	5.0	5.0	1.0	1.0	4.0	4.0
	Aspirants	5.0	4.0	2.0	3.0	2.0	2.0	5.0	5.0	1.0	2.0	1.0	1.0	7.0	5.0	1.0	1.0	1.0	2.0
	Enthusiasts	6.0	5.0	3.0	3.0	3.0	3.0	5.0	5.0	2.0	2.0	1.0	1.0	6.0	5.0	1.0	1.0	3.0	2.0
Sparkling white (MT) (*n* = 132)	All Consumers	6.0	3.0	3.0	2.0	3.0	2.0	6.5	3.0	3.0	1.5	1.0	1.0	7.0	3.0	1.0	1.0	3.0	2.0
	No Frills	7.0	5.0	2.0	2.0	3.0	2.0	5.0	5.0	3.0	2.0	1.0	1.0	7.0	5.0	1.0	2.0	3.0	3.0
	Aspirants	5.0	3.0	3.0	2.0	3.0	2.0	6.0	3.0	2.0	2.0	2.0	1.0	7.0	3.0	1.0	1.0	5.0	2.0
	Enthusiasts	7.0	2.0	3.5	2.5	3.5	2.0	7.0	3.0	3.0	1.0	1.0	1.0	7.0	3.0	1.0	1.0	2.5	2.0
Sparkling rosé (*n* = 139)	All Consumers	2.0	4.0	1.0	2.0	1.0	2.0	3.0	5.0	1.0	2.0	1.0	1.0	3.0	5.0	1.0	1.0	1.0	2.0
	No Frills	1.0	3.0	1.0	3.0	1.0	1.0	2.0	3.0	1.0	1.0	1.0	1.0	2.0	3.0	1.0	1.0	1.0	2.0
	Aspirants	2.0	4.0	2.0	2.0	1.0	2.0	3.0	5.0	1.0	2.0	1.0	1.0	3.0	5.0	1.0	1.0	1.0	2.0
	Enthusiasts	1.0	5.0	1.0	2.0	1.0	2.0	3.0	5.0	1.0	2.0	1.0	1.0	3.0	5.0	1.0	1.0	1.0	3.0
Sparkling red (*n* = 130)	All Consumers	3.0	3.0	1.0	2.0	1.0	2.0	2.0	5.0	1.0	2.0	1.0	1.0	4.0	5.0	1.0	1.0	1.0	2.0
	No Frills	1.0	1.0	1.0	3.0	1.0	1.0	2.0	3.0	1.0	1.0	1.0	1.0	2.0	2.0	1.0	1.0	1.0	1.0
	Aspirants	3.0	3.0	2.0	2.0	1.0	2.0	3.0	5.0	1.0	2.0	1.0	2.0	5.0	4.0	1.0	1.0	1.0	2.0
	Enthusiasts	3.0	4.0	1.0	2.0	1.0	2.0	3.0	5.0	1.0	2.0	1.0	1.0	4.0	5.0	1.0	1.0	1.0	2.5
Moscato (*n* = 137)	All Consumers	1.0	2.0	1.0	1.0	1.0	1.0	1.0	2.0	1.0	1.0	1.0	1.0	1.0	3.0	1.0	1.0	1.0	1.0
	No Frills	1.0	1.0	1.0	1.0	1.0	1.0	1.0	1.0	1.0	1.0	1.0	1.0	1.0	2.0	1.0	1.0	1.0	1.0
	Aspirants	1.0	2.0	1.0	1.0	1.0	1.0	1.0	3.0	1.0	1.0	1.0	1.0	1.5	3.0	1.0	1.0	1.0	1.0
	Enthusiasts	1.0	2.0	1.0	1.0	1.0	1.0	1.0	1.5	1.0	1.0	1.0	1.0	1.0	2.0	1.0	1.0	1.0	1.0
Prosecco (*n* = 138)	All Consumers	1.0	3.0	1.0	2.0	1.0	2.0	1.0	4.0	1.0	2.0	1.0	1.0	1.0	4.0	1.0	1.0	1.0	2.0
	No Frills	1.0	3.0	1.0	1.5	1.0	1.5	1.0	3.0	1.0	1.5	1.0	1.0	1.0	3.0	1.0	1.0	1.0	2.5
	Aspirants	1.0	4.0	1.0	2.0	1.0	1.0	1.0	4.0	1.0	1.0	1.0	1.0	1.0	4.0	1.0	1.0	1.0	2.0
	Enthusiasts	1.0	3.5	1.0	3.0	1.0	2.0	1.0	4.0	1.0	2.0	1.0	1.0	1.0	5.0	1.0	1.0	1.0	2.0

		Hot weather		Melbourne Cup		New Year		Pub or club		Restaurant or café		Weddings		Weekdays		Weekends		Work drinks	
Champagne (*n* = 137)	All Consumers	3.0	3.0	3.0	3.0	7.0	5.0	1.0	2.0	3.0	4.0	7.0	5.0	2.0	2.0	4.0	4.0	2.0	3.0
	No Frills	2.0	3.0	1.0	5.0	7.0	6.0	2.0	3.0	5.0	5.0	6.0	5.0	2.0	3.0	4.0	5.0	3.0	4.0
	Aspirants	3.0	3.0	2.0	3.0	7.0	5.0	1.0	2.0	3.0	4.0	7.0	5.0	2.0	2.0	3.0	5.0	1.0	3.0
	Enthusiasts	3.0	5.0	5.0	4.0	7.0	5.0	2.0	2.0	3.0	5.0	7.0	5.0	2.0	2.0	5.0	3.0	2.0	3.0
Sparkling white (MT) (*n* = 132)	All Consumers	5.0	2.0	5.0	3.0	7.0	3.0	3.0	2.0	5.0	3.0	7.0	3.0	3.0	2.0	5.0	3.0	3.0	2.0
	No Frills	3.0	3.0	5.0	3.0	7.0	5.0	3.0	2.0	5.0	5.0	7.0	5.0	3.0	3.0	5.0	5.0	3.0	5.0
	Aspirants	5.0	2.0	3.0	2.0	7.0	3.0	3.0	2.0	5.0	3.0	7.0	3.0	2.0	2.0	5.0	3.0	3.0	2.0
	Enthusiasts	5.0	3.0	5.0	2.0	8.0	3.0	2.0	2.0	5.0	3.0	7.0	3.0	3.0	1.5	6.0	3.0	3.0	2.0
Sparkling rosé (*n* = 139)	All Consumers	2.0	3.0	1.0	3.0	2.0	5.0	1.0	2.0	2.0	3.0	2.0	5.0	1.0	2.0	2.0	3.0	1.0	3.0
	No Frills	1.0	1.0	1.0	2.0	1.0	3.0	1.0	2.0	3.0	3.0	1.0	3.0	1.0	2.0	1.0	3.0	1.0	2.0
	Aspirants	3.0	3.0	1.0	3.0	2.0	5.0	1.0	2.0	2.0	4.0	2.0	5.0	1.0	2.0	2.0	3.0	1.0	3.0
	Enthusiasts	2.0	3.0	1.0	4.0	2.0	5.0	1.0	2.0	3.0	5.0	2.0	5.0	1.0	2.0	1.0	5.0	1.0	3.0
Sparkling red (*n* = 130)	All Consumers	2.0	3.0	1.0	2.0	2.0	5.0	1.0	2.0	3.0	4.0	2.0	5.0	1.0	2.0	2.0	3.0	1.0	2.0
	No Frills	1.0	1.0	1.0	1.0	1.0	3.0	1.0	1.0	2.0	3.0	1.0	3.0	1.0	2.0	1.0	3.0	1.0	2.0
	Aspirants	3.0	3.0	1.0	2.0	2.0	5.0	2.0	2.0	3.0	3.0	2.0	5.0	1.0	2.0	2.0	3.0	1.0	3.0
	Enthusiasts	2.0	4.0	1.0	2.5	2.0	5.0	1.0	2.0	3.0	5.0	2.0	5.0	1.0	2.0	1.0	5.0	1.0	2.0
Moscato (*n* = 137)	All Consumers	1.0	2.0	1.0	1.0	1.0	2.0	1.0	1.0	1.0	2.0	1.0	3.0	1.0	1.0	1.0	2.0	1.0	1.0
	No Frills	1.0	1.0	1.0	1.0	1.0	2.0	1.0	1.0	1.0	1.0	1.0	2.0	1.0	1.0	1.0	1.0	1.0	1.0
	Aspirants	1.0	2.0	1.0	1.0	1.0	3.0	1.0	1.0	1.0	2.0	1.0	3.0	1.0	1.0	1.0	2.0	1.0	2.0
	Enthusiasts	1.0	2.0	1.0	1.0	1.0	2.0	1.0	1.0	1.0	2.0	1.0	2.0	1.0	1.0	1.0	2.0	1.0	1.0
Prosecco (*n* = 138)	All Consumers	1.0	3.0	1.0	3.0	1.0	4.0	1.0	2.0	1.0	3.0	1.0	4.0	1.0	2.0	1.0	3.0	1.0	3.0
	No Frills	1.0	1.5	1.0	3.0	1.0	3.0	1.0	2.5	1.0	3.0	1.0	4.5	1.0	2.5	1.0	3.0	1.0	3.0
	Aspirants	1.0	2.0	1.0	3.0	1.0	4.0	1.0	2.0	1.0	3.0	1.0	4.0	1.0	2.0	1.0	3.0	1.0	2.0
	Enthusiasts	1.0	3.0	1.0	3.0	1.0	4.0	1.0	1.5	1.0	5.0	1.0	4.0	1.0	2.0	1.0	4.0	1.0	3.0

Data represent medians of 9-point Likert scale scores (where 1 = never consume, 5 = sometimes consume, and 9 = always consume).

Table S5. Statistical analysis for influence of occasion on consumers' and Fine Wine Instrument consumer segments' consumption of different sparkling wine styles.

		Survey Mood's Median Test Multiple Pairwise P-value | Tasting Mood's Median Test Multiple Pairwise P-value																	
		**Anniversary**		**At home with food**		**At home without food**		**Birthday**		**Breakfast**		**By yourself**		**Christmas**		**Funeral**		**Girls' or boys' night**	
Champagne (n = 137)	All Segments	0.277	0.505	0.682	0.820	0.593	0.909	0.931	0.488	0.312	0.788	0.152	0.784	0.779	0.519	0.773	0.655	0.188	0.116
	No Frills vs Aspirants	0.135	0.318	0.982	0.635	0.676	0.715	0.748	0.355	0.789	0.741	0.210	0.899	0.479	0.904	0.674	0.493	0.083	0.038*
	Aspirants vs Enthusiasts	0.849	0.398	0.383	0.781	0.599	0.744	0.911	0.324	0.140	0.501	0.073	0.528	0.849	0.258	0.648	0.436	0.311	0.528
	No Frills vs Enthusiasts	0.127	0.649	0.685	0.528	0.311	0.382	0.708	0.840	0.342	0.890	0.961	0.592	0.390	0.529	0.492	0.907	0.540	0.198
Sparkling white (MT) (n = 132)	All Segments	0.481	0.949	0.585	0.379	0.817	0.789	0.501	0.324	0.195	0.926	0.362	0.949	0.407	0.213	0.570	0.292	0.200	0.594
	No Frills vs Aspirants	0.712	0.835	0.764	0.400	0.877	0.638	0.803	0.542	0.394	0.835	0.258	0.062	0.657	0.211	0.764	0.139	0.803	0.322
	Aspirants vs Enthusiasts	0.278	0.763	0.395	0.186	0.590	0.741	0.762	0.262	0.085	0.756	0.242	0.763	0.180	0.427	0.289	0.986	0.073	0.944
	No Frills vs Enthusiasts	0.035*	0.980	0.368	0.857	0.590	0.492	0.656	0.159	0.829	0.726	0.829	0.013*	0.575	0.078	0.617	0.160	0.386	0.378
Sparkling rosé (n = 139)	All Segments	0.380	0.040*	0.448	0.905	0.631	0.848	0.778	0.076	0.226	0.479	0.795	0.223	0.357	0.223	0.683	0.702	0.714	0.709
	No Frills vs Aspirants	0.774	0.057	0.281	0.721	0.426	0.571	0.505	0.023*	0.256	0.232	0.546	0.087	0.444	0.035*	0.668	0.564	0.452	0.571
	Aspirants vs Enthusiasts	0.218	0.473	0.355	0.871	0.473	0.957	0.518	0.762	0.329	0.605	0.882	0.871	0.176	0.871	0.534	0.459	0.961	0.561
	No Frills vs Enthusiasts	0.915	0.013*	0.710	0.657	0.800	0.624	0.511	0.047*	0.092	0.444	0.506	0.135	0.822	0.127	0.425	0.978	0.438	0.409
Sparkling red (n = 130)	All Segments	0.204	0.088	0.513	0.103	0.485	0.322	0.127	0.015*	0.068	0.107	0.818	0.039*	0.258	0.319	0.259	0.154	0.530	0.815
	No Frills vs Aspirants	0.077	0.206	0.316	0.166	0.258	0.312	0.043*	0.247	0.820	0.091	0.616	0.011*	0.101	0.166	0.107	0.053	0.381	0.532
	Aspirants vs Enthusiasts	0.508	0.158	0.834	0.302	0.722	0.469	0.434	0.032*	0.039*	0.496	0.601	0.342	0.669	0.608	0.487	0.588	0.704	0.944
	No Frills vs Enthusiasts	0.111	0.034*	0.261	0.035*	0.235	0.134	0.156	0.008*	0.103	0.035*	0.916	0.069	0.111	0.130	0.241	0.130	0.261	0.284
Moscato (n = 137)	All Segments	0.143	0.166	0.109	0.880	0.220	0.526	0.206	0.038*	0.946	0.198	0.049*	0.394	0.178	0.046*	0.377	0.551	0.563	0.845
	No Frills vs Aspirants	0.395	0.129	0.084	0.852	0.085	0.229	0.358	0.035*	0.756	0.079	0.677	0.193	0.204	0.581	0.985	0.769	0.288	0.572
	Aspirants vs Enthusiasts	0.673	0.128	0.118	0.615	0.520	0.526	0.086	0.044*	0.978	0.405	0.014*	0.461	0.096	0.013*	0.173	0.301	0.677	0.767
	No Frills vs Enthusiasts	0.759	0.173	0.590	0.840	0.217	0.502	0.661	0.612	0.755	0.278	0.080	0.478	0.955	0.717	0.227	0.136	0.478	0.757
Prosecco (n = 138)	All Segments	0.033*	0.916	0.050	0.461	0.653	0.203	0.025*	0.798	0.096	0.490	0.115	0.986	0.018*	0.305	0.010*	0.345	0.132	0.316
	No Frills vs Aspirants	0.009*	0.679	0.029*	0.613	0.131	0.949	0.006*	0.716	0.031*	0.949	0.055	0.905	0.005*	0.373	0.041*	0.804	0.052	0.164
	Aspirants vs Enthusiasts	0.501	0.609	0.588	0.641	0.069	0.093	0.549	0.500	0.971	0.994	0.678	0.943	0.442	0.354	0.018*	0.150	0.347	0.284
	No Frills vs Enthusiasts	0.030*	0.739	0.014*	0.137	0.024*	0.867	0.021*	0.867	0.034*	0.289	0.034*	0.866	0.021*	0.289	0.476	0.381	0.185	0.616

		**Hot weather**		**Melbourne Cup**		**New Year**		**Pub or club**		**Restaurant or café**		**Weddings**		**Weekdays**		**Weekends**		**Work drinks**	
Champagne (n = 137)	All Segments	0.312	0.300	0.161	0.496	0.999	0.434	0.040*	0.149	0.434	0.765	0.892	0.651	0.445	0.393	0.203	0.412	0.803	0.889
	No Frills vs Aspirants	0.986	0.538	0.904	0.392	0.977	0.205	0.290	0.053	0.205	0.576	0.787	0.355	0.986	0.205	0.576	0.232	0.510	0.644
	Aspirants vs Enthusiasts	0.140	0.124	0.069	0.311	0.974	0.553	0.012*	0.756	0.927	0.548	0.651	0.841	0.218	0.400	0.075	0.381	0.804	0.908
	No Frills vs Enthusiasts	0.342	0.267	0.204	0.504	0.961	0.437	0.382	0.111	0.265	0.484	0.961	0.483	0.429	0.540	0.110	0.839	0.653	0.653
Sparkling white (MT) (n = 132)	All Segments	0.994	0.189	0.778	0.548	0.551	0.044*	0.671	0.254	0.713	0.046*	0.375	0.009*	0.229	0.080	0.150	0.026*	0.642	0.028*
	No Frills vs Aspirants	0.453	0.107	0.803	0.399	0.638	0.218	0.395	0.400	0.878	0.061	0.835	0.009*	0.211	0.033*	0.561	0.024*	0.727	0.062
	Aspirants vs Enthusiasts	0.973	0.188	0.826	0.812	0.278	0.427	0.615	0.272	0.415	0.333	0.211	0.379	0.158	0.958	0.051	0.468	0.468	0.039*
	No Frills vs Enthusiasts	0.936	0.811	0.617	0.278	0.829	0.013*	0.656	0.105	0.656	0.013*	0.273	0.003*	0.143	0.051	0.465	0.008*	0.386	0.013*
Sparkling rosé (n = 139)	All Segments	0.592	0.086	0.179	0.013*	0.531	0.065	0.522	0.921	0.766	0.232	0.756	0.129	0.719	0.640	0.712	0.055	0.763	0.121
	No Frills vs Aspirants	0.505	0.080	0.332	0.014*	0.319	0.105	0.365	0.865	0.574	0.091	0.200	0.045*	0.486	0.574	0.498	0.187	0.636	0.098
	Aspirants vs Enthusiasts	0.355	0.381	0.201	0.341	0.804	0.218	0.354	0.756	0.548	0.744	0.600	0.651	0.561	0.561	0.530	0.108	0.501	0.441
	No Frills vs Enthusiasts	0.986	0.027*	0.082	0.003*	0.272	0.021*	0.849	0.710	0.203	0.153	0.227	0.046*	0.800	0.352	0.915	0.021*	0.978	0.040*
Sparkling red (n = 130)	All Segments	0.600	0.138	0.096	0.255	0.029*	0.063	0.258	0.149	0.552	0.351	0.201	0.255	0.816	0.485	0.204	0.323	0.750	0.675
	No Frills vs Aspirants	0.529	0.315	0.053	0.821	0.008*	0.206	0.101	0.053	0.454	0.710	0.077	0.206	0.529	0.312	0.023*	0.454	0.622	0.448
	Aspirants vs Enthusiasts	0.443	0.109	0.737	0.144	0.463	0.348	0.044*	0.749	0.323	0.231	0.763	0.551	0.763	0.772	0.508	0.326	0.704	0.492
	No Frills vs Enthusiasts	0.362	0.061	0.035*	0.200	0.061	0.023*	0.261	0.080	1.000	0.219	0.134	0.098	0.698	0.235	0.206	0.151	0.405	0.819
Moscato (n = 137)	All Segments	0.367	0.720	0.982	0.769	0.125	0.280	0.673	0.643	0.360	0.403	0.268	0.378	0.194	0.720	0.491	0.890	0.887	0.518
	No Frills vs Aspirants	0.348	0.423	0.978	0.503	0.367	0.038*	0.377	0.348	0.941	0.431	0.348	0.880	0.125	0.423	0.423	0.753	0.803	0.508
	Aspirants vs Enthusiasts	0.200	0.723	0.863	0.951	0.785	0.494	0.901	0.767	0.166	0.201	0.125	0.994	0.189	0.723	0.280	0.656	0.635	0.273
	No Frills vs Enthusiasts	0.940	0.625	0.876	0.502	0.348	0.160	0.453	0.506	0.320	0.824	0.785	0.112	0.610	0.625	0.955	0.969	0.902	0.840
Prosecco (n = 138)	All Segments	0.050*	0.124	0.164	0.389	0.024	0.615	0.069	0.533	0.017*	0.128	0.008*	0.946	0.027*	0.067	0.010*	0.219	0.187	0.221
	No Frills vs Aspirants	0.029*	0.526	0.090	0.854	0.006*	0.324	0.039*	0.265	0.005*	0.934	0.002*	0.747	0.010*	0.068	0.003*	0.406	0.069	0.504
	Aspirants vs Enthusiasts	0.588	0.063	0.237	0.180	0.721	0.721	0.153	0.609	0.771	0.061	0.237	0.853	0.813	0.042*	0.994	0.243	0.868	0.082
	No Frills vs Enthusiasts	0.014*	0.128	0.366	0.402	0.014*	0.317	0.261	0.502	0.009*	0.134	0.016*	0.868	0.007*	0.868	0.003*	0.095	0.092	0.494

* denotes P values at ≤ 0.05.
